# A human transcription factor in search mode

**DOI:** 10.1093/nar/gkv1091

**Published:** 2015-12-15

**Authors:** Kevin Hauser, Bernard Essuman, Yiqing He, Evangelos Coutsias, Miguel Garcia-Diaz, Carlos Simmerling

**Affiliations:** 1 Laufer Center for Physical and Quantitative Biology, Stony Brook University, Stony Brook, NY 11794, USA; 2 Department of Chemistry, Stony Brook University, Stony Brook, NY 11794, USA; 3 Suffolk Community College, Selden, NY 11784, USA; 4 Great Neck South High School, Great Neck, NY 11023, USA; 5 Department of Applied Mathematics and Statistics, Stony Brook University, Stony Brook, NY 11794, USA; 6 Department of Pharmacological Sciences, Stony Brook University, Stony Brook, NY 11794, USA

## Abstract

Transcription factors (TF) can change shape to bind and recognize DNA, shifting the energy landscape from a weak binding, rapid search mode to a higher affinity recognition mode. However, the mechanism(s) driving this conformational change remains unresolved and in most cases high-resolution structures of the non-specific complexes are unavailable. Here, we investigate the conformational switch of the human mitochondrial transcription termination factor MTERF1, which has a modular, superhelical topology complementary to DNA. Our goal was to characterize the details of the non-specific search mode to complement the crystal structure of the specific binding complex, providing a basis for understanding the recognition mechanism. In the specific complex, MTERF1 binds a significantly distorted and unwound DNA structure, exhibiting a protein conformation incompatible with binding to B-form DNA. In contrast, our simulations of apo MTERF1 revealed significant flexibility, sampling structures with superhelical pitch and radius complementary to the major groove of B-DNA. Docking these structures to B-DNA followed by unrestrained MD simulations led to a stable complex in which MTERF1 was observed to undergo spontaneous diffusion on the DNA. Overall, the data support an MTERF1-DNA binding and recognition mechanism driven by intrinsic dynamics of the MTERF1 superhelical topology.

## INTRODUCTION

One in ten genes in the human genome encodes a transcription factor (TF) (1), and once expressed, TFs direct the expression of other genes. TFs adapt conformation to switch function: to bind, search or recognize DNA. To rapidly respond to stimulus, TFs must locate target DNA quickly. Three dimensional (3D) diffusion from solution directly onto the target DNA site, amongst an excess of non-specific sites, predicts on-rates 10-fold slower than observed *in vivo* (2). Thus, 3D diffusion and 1D facilitated diffusion (sliding) likely drive target search (3–8). Frustration during 1D diffusion can arise when affinity for non-specific DNA is high. Theory predicted (7,8) and experiments on p53 corroborated (9,10) that TFs most likely switch from a rapid search mode to a tight-binding recognition mode by changing conformation. In search mode, scanning is facilitated by fleeting non-specific binding with ∼1 *k*_B_T energy gaps that reduce residence time on non-cognate sites (8). Significant perturbation of the DNA structure is unlikely on such small energy and time scales. Thus, a transcription factor should be able to weakly bind a random sequence of DNA, that is presumably B-form (11,12).

### Conformational change regulates recognition

During recognition the TF can conformationally adapt to optimize specific contacts that directly recognize chemical groups present in the cognate sequence, shifting the free energy landscape to a regime with large energy gaps and high barriers between specific and non-specific sites (8). The kinetic aspect of recognition is analogous to enzyme inhibitors that exhibit long residence times following an induced fit conformational change in the protein (13,14). Dynamics of the tightly bound TF can also induce DNA deformation, potentially giving rise to dynamic indirect readout via sequence-dependent deformability of DNA, or to shape readout (static indirect readout) (15–17). Therefore, conformational changes in the TF and in the DNA during recognition are coupled dynamic processes that depend on atomistic intermolecular interactions—direct readout—and intramolecular interactions—indirect readout—and protein strain. For example, NMR transverse relaxation rate measurements of the lac repressor headpiece reveal that amino acids involved in direct readout in the recognition mode form non-specific interactions with the phosphate backbone in the search mode (18). The data suggest that conformational adaptation from search to recognition modes includes switching non-specific contacts with the DNA backbone to specific TF-nucleobase interactions. TF–DNA binding and recognition is thus a function of the relative energies of the search and recognition metastates, which is determined by the thermodynamics and kinetics of TF and DNA conformational change.

The relative importance, however, of direct and indirect readout during the transition from search to recognition mode is poorly understood. Insight into the mechanism of conformational change, and thus of recognition, would be facilitated by high resolution structural data for specific and non-specific complexes. The lac repressor headpiece (18) and the enzymes BamHI (19), BstYI (20) and EcoRV (21) are prototypical DNA-binding proteins for which static structures of specific and putative non-specific complexes have been experimentally characterized. However, the lifetime of a true non-specific complex is by definition fleeting (8). To favour binding at a single non-specific site requires alterations to the DNA or protein, truncated constructs or protein–DNA cross-links that artificially stabilize the energy of the non-specific complex. In these altered complexes, usually only a few interactions have been modified and therefore a subset of the cognate recognition contacts may still be present—‘hemispecific recognition’ (20)—and the DNA is frequently shifted from B-form. For example, the structure of the human transcription factor MTERF1 was solved for a putative non-specific complex in which a subset of the recognition interactions were eliminated. The DNA conformation was deformed, however, and resembled that seen in the fully cognate complex (22); the DNA conformation in putative non-specific complexes of BamHI (23), BstYI (20) and EcoRV (24) enzymes also resemble that in the cognate complex. Consequently, it is unclear how accurately these altered complexes represent the actual structure during rapid search, outside the influence of methods used to redirect binding specificity and trap a unique non-cognate structure. Moreover, static snapshots do not resolve dynamics. A complete mechanistic picture of how TFs regulate gene expression would involve a dynamic model of the ensemble of structures that correspond to search mode, as well as an atomistic description of the conformational and energetic changes that take place during the transition from non-specific to specific complexes. Here, we use a combination of experimental structural data and molecular dynamics simulations to address the first element in this challenge and develop a dynamic model for non-specific DNA binding, using as a model system the human mitochondrial transcription factor MTERF1.

The Mitochondrial TERmination Factors (MTERFs) are vital TFs (22). MTERFs are involved in regulating gene expression in the mitochondria of eukaryotes and also the plastids of plants (25). MTERF1 is the canonical mitochondrial transcription terminator, responsible for modulating the expression of mitochondrial DNA (mtDNA) genes (26) by preventing L-strand transcription interference (27) within the circular mtDNA. Mitochondrial dysfunction resulting from alterations in mitochondrial gene expression has been correlated with ageing, cancer, diabetes, and neurological disorders like Parkinson's disease and Alzheimer's disease (28,29). Since MTERF proteins play essential roles in mediating gene expression in mitochondria and chloroplasts, further understanding the mechanism by which MTERF1 interacts with DNA will contribute to our understanding of organellar biology and the connection between bioenergy and disease. Furthermore, defects in MTERF1 binding have been previously associated with mitochondrial disease (22,30–31).

MTERF1 has a modular tertiary structure topology. Modularity in TF tertiary structure is important for combinatorial discretization of binding site specificity and evolutionary stability (32), possibly explaining the abundance of organellar TFs that are modular (33). The TAL effector is another superhelical TF whose modular structure eases the retargeting of specificity for genome editing (34). Park *et al*. showed that it is possible to customize macromolecular topologies by mixing and matching leucine-rich repeat modules (35). Overall, modularity simplifies the challenge of characterizing the mechanism of protein–DNA search and recognition because modules can act as small and independent but linked proteins, thereby reducing the mechanical degrees of freedom likely to be important for functional dynamics.

**Figure 1.**
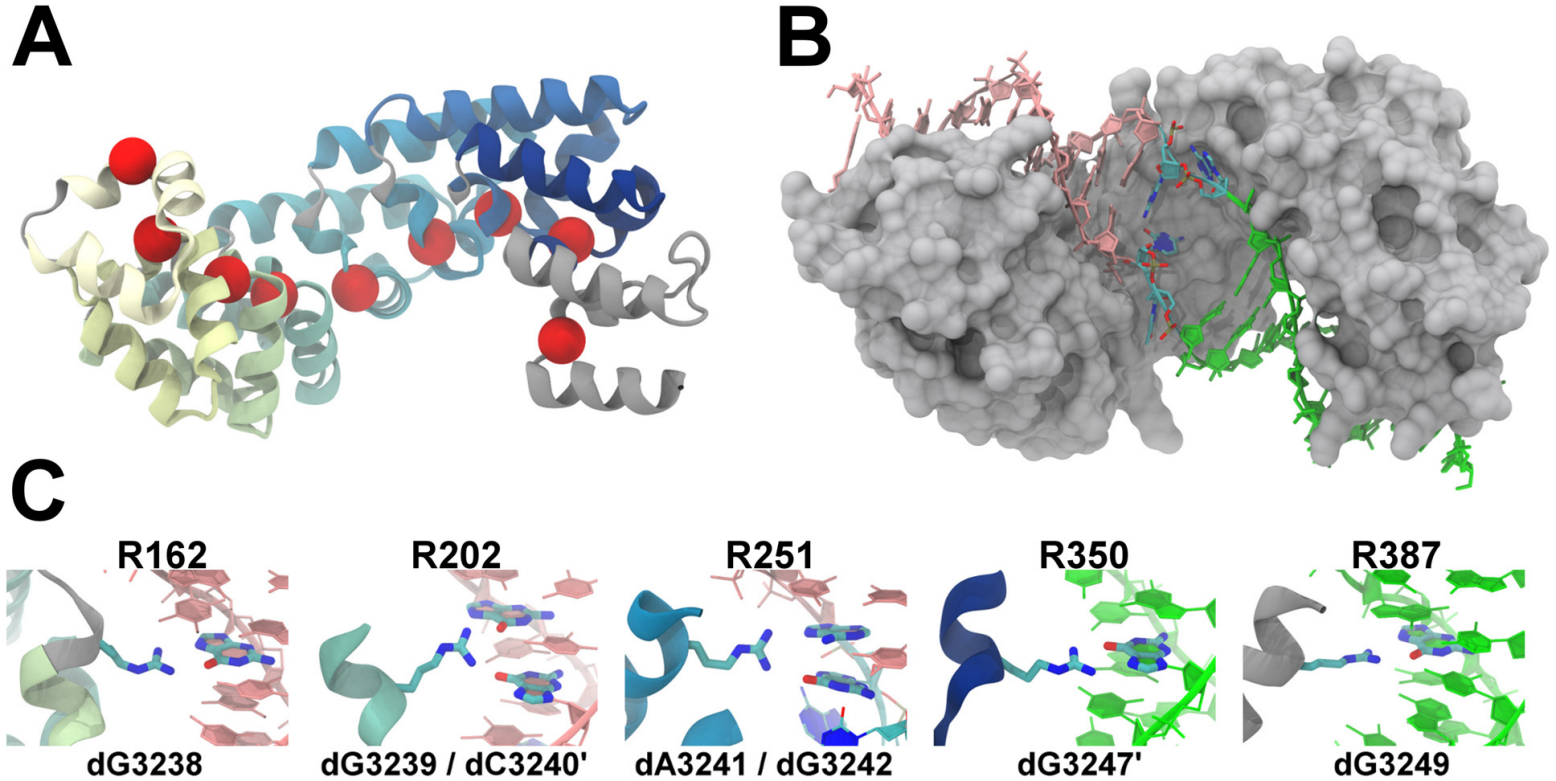
Human MTERF1 is a modular, superhelical TF that unwinds target DNA in recognition mode. (**A**) MTERF1 is composed of 8 *mterf* motifs (coloured from yellow to blue). Intervening S-loops and the C-segment are grey. Superhelical residues are shown as red spheres. (**B**) The superhelical topology of MTERF1 (grey MSMS surface (81)) tracks the major groove of DNA. The bound DNA (displayed as sticks and ribbons) is unwound, which is focused on the central two base pairs (coloured by element), while the N-site of the DNA (pink) and the C-site of the DNA (green) remain essentially undeformed. (**C**) MTERF1 forms direct readout interactions in the N-site and C-site: R162 forms a double H-bond with the N7 and O6 of dG3238 (light-strand, LS); R202 bridges a cross-strand dinucleotide step, H-bonding with O6 of dG3239 (LS) and dG3240’ (heavy-strand, HS); R251 bridges a dinucleotide step on the HS, H-bonding to the N7 of dA3241 and O6 of dG3242; R350 double H-bonds with the N7 and O6 of dG3247’ (LS); R387 double H-bonds with dG3249.

The X-ray crystal structure of MTERF1 bound to the cognate termination sequence was resolved at 2.20 Å (22), revealing a superhelical topology (36) complementary to the bound DNA structure (Figure 1). The apparent architectural complementarity of MTERF1 and DNA simplifies the structure-dynamics-function relationship in MTERF1–DNA binding. MTERF1 is modular, composed of eight 33-residue *mterf* motifs (22) that represent steps in the superhelix (Figure 1A). Each *mterf* motif is composed of a triangular arrangement of three short helices stabilized by a hydrophobic core. Fewer packing interactions between motifs suggests that local changes in motif–motif stacking could give rise to global dynamics in superhelical pitch and radius. The macrodipole of the central helix in motifs 5 through 8 align with the DNA phosphate backbone. Capping the central α-helices in all 8 motifs are conserved proline residues, creating an S-loop that prevents a steric clash with the DNA.

Unwinding of the bound DNA is dramatic (Figure 1B), providing structural support for a roadblock termination mechanism (22,37). The unwinding induced by MTERF1 is focused on the central two base pairs (Figure 1B), which are everted from the duplex and stabilized by hydrogen bonds and stacking interactions. On either side of the flipped bases the DNA is essentially B-form, but the helical axis is bent ∼30° over the everted bases (Supplementary Figure S1). Importantly, although the DNA is unwound, MTERF1 tracks the major groove across the full 22 bp footprint. The conserved proline residues within each motif line the major groove; tracing their path outlines the superhelical topology of MTERF1 (Figure 1A) and the complementarity to the unwound DNA (Figure 1B). MTERF1 forms direct readout interactions with the bases in the B-form N-site and C-site segments of DNA, presumably stabilizing the intervening distortion in the duplex (Figure 1C).

**Figure 2.**
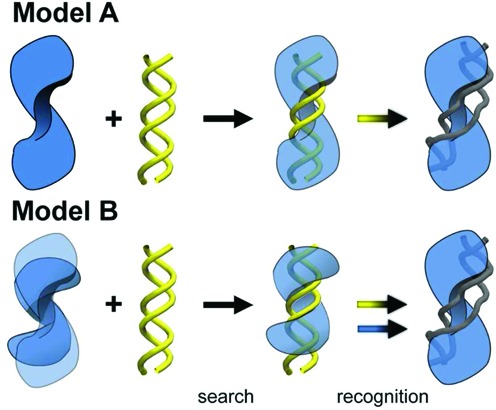
Potential MTERF1–DNA binding and recognition mechanisms. Pre-existing unwound DNA that MTERF1 can bind is not likely a viable mechanism (see text). We consider models with either singly or doubly induced fit. **Model A**: MTERF1 (blue) undergoes minimal conformational adaptation during binding and recognition, with the structure of apo MTERF1, MTERF1 in search mode (non-specific complex) bound to B-DNA (yellow), and MTERF1 in recognition mode bound to unwound DNA (grey) all being similar in structure. During recognition, only the DNA undergoes conformational change (yellow arrow). **Model B**: apo MTERF1 is flexible, sampling a diverse ensemble of structures including those with a helical topology similar to B-DNA. During doubly induced fit recognition, both MTERF1 and DNA undergo conformational change, blue and yellow arrows, respectively.

Representing a key knowledge gap in MTERF biology, neither the apo mode nor the search mode of MTERF1 has been structurally characterized. What might the conformations of apo MTERF1 and the MTERF1–DNA non-specific complex be? MTERF1 binding to a transiently unwound DNA duplex seems unlikely since similar DNA deformation in EcoRI was estimated to cost ∼100 kcal/mol in strain and entropy (12). We explore two potentially more reasonable models in Figure 2. First, the conformation of holo and apo MTERF1 might be similar, as observed for BamHI (19), implying MTERF1 binds B-DNA and only the DNA changes conformation during recognition (Figure 2, **Model A**). However, we show below that the conformation of MTERF1 observed in the specific complex cannot accommodate B-form DNA without extensive steric clashes, as one might presume given the high level of DNA distortion observed in the complex. Alternatively, apo MTERF1 may be capable of adapting conformation to bind B-form DNA via induced fit (38), following a fly-casting binding mechanism (39), in which an unstructured tail increases the protein–DNA collision radius, or a gated binding mechanism (40), in which the protein oscillates to admit or deny ligand (DNA) entry into the binding pocket (Figure 2, **Model B**). Attempts to crystallize the protein in the absence of DNA were unsuccessful (Garcia–Diaz, unpublished data), supporting our hypothesis that apo MTERF1 is flexible or locally unstructured similar to p53 (41), lac repressor headpiece (18) and the tails of SRY (42). The difference between these models lies largely in the extent to which dynamics of MTERF1 plays a role in DNA binding and recognition.

While p53, lac repressor headpiece, and SRY have been extensively studied in the literature, how any TF undergoes a search to recognition conformational switch remains a gap in our knowledge. What might the conformational switch be for MTERF1? We hypothesize that subsequent to sliding to the target, unpacking of the central *mterf* motifs near the flipped bases might accompany DNA unwinding during recognition, allowing the superhelical pitch of the TF to adapt to, or perhaps drive, distortion in the curvature of the major groove during unwinding. Molecular simulations have been used in the past to study the role of flexibility in protein–DNA recognition (43–46) and inhibition (47). Here, we report results of coarse grain elastic network model calculations as well as μs-timescale atomistic molecular dynamics (MD) simulations. Despite fundamental differences in the methods, both approaches support the same conclusion that the superhelical topology of MTERF1 is dynamic. The ensemble of structures obtained in MD samples a broad range of superhelical pitch and radius, including conformations matching the corresponding pitch and radius of B-DNA. Docking these low pitch apo MTERF1 structures to a B-DNA duplex resulted in a stable, dynamic complex in which MTERF1 shows 1D diffusion along the major groove of B-like DNA, providing an atomic resolution, dynamic model for a model TF searching DNA.

## MATERIALS AND METHODS

### Helix analysis

Calculation of pitch and radius of MTERF1 used the Cartesian coordinates of the Cα atoms in positions that most closely track the major groove of DNA. The steps along the helix were defined by the Cα atoms of the S-loop forming prolines, with two exceptions. First for motif 6, the Cα of A279 was used instead of P277 since the distances between the Cα atoms in motifs 5 and 6 and between motifs 6 and 7 are significantly larger and smaller than other steps, respectively. The distinctive geometry of motifs 5, 6, 7 that track the unwound central site of DNA is likely related to how MTERF1 unwinds DNA. Also, P277 of motif 6 is in a GPG loop, the flexibility of which might potentially lead to local changes that could affect measurement of global dynamics. Second, the Cα of W383 was used in the C-segment (Figure 1A), which lacks a proline residue. The positions of the superhelical residues are shown in Figure 1A.

The Cα coordinates for the nine superhelical residues (Figure 1A) were projected onto a rotatable plane using Supplementary Equation S1 to find the plane that contained the best circle according to a linear least squares procedure solved by singular value decomposition (Supplementary Section 2). The radius of the circle (helix radius) on the rotated plane was obtained directly from the fitting solution (Supplementary Equation S2). With respect to the rotated plane and its frame, the sum of the angles swept between consecutive Cα atoms (8 angles between 9 helical steps) gives the helical sweep Φ. The superhelical pitch, *κ*, is then the distance between the first and last atom (e.g. Cα_1_ and Cα_9_) along the helical axis, Δz, multiplied by Φ/2π. The ensemble of apo MTERF1 structures was aligned, an unconstrained grid search (in *φ* and *θ*) was performed for all structures, and the region of the grid that resulted in low fit residuals was determined, *φ* in [50°,70°] and *θ* in [240°,300°] (Supplementary Figure S2).

### Anisotropic network model

Using ProDy (48), the anisotropic network model (ANM) modes were calculated using the crystallographic coordinates of MTERF1 Cα atoms (PDB: 3MVA) (22) with a distance weight of 2.5 (49). A cutoff of 24 Å was selected because it gave the best correlation to B-factors (see Supplementary Section 3). To display structures projected along the unit modes, a factor of 50 was used to arbitrarily scale up the displacements. To compare ANM and MD results, the overlap of the eigenvectors obtained from each method was calculated. The root mean square inner product (RMSIP) was used to compare all pairs of ANM and MD eigenvectors. (50).

### Model building and parameter preparation

#### Specific MTERF1–DNA complex

Coordinates were obtained from the crystal structure of MTERF1 bound to DNA (PDB code 3MVA) (22). Density was missing for a disordered 19 residue N-terminal segment and the side chains on the first two N-terminal residues of the resolved chain (residues 20 and 21). The role of the disordered segment in binding and recognition was beyond the scope of this work (perhaps involved in signalling, part of the mitochondrial targeting sequence, etc.) and was removed from the model. The sidechains of residues 20 and 21 were added using Amber libraries (51). One hundred and eighty eight water O atoms were resolved and retained in our model building. Molprobity (52) was used to add H atoms to the model and check for N/Q/H flips; none were strongly favoured over the original model. The complex was then encapsulated in a 96.3 Å truncated octahedron of explicit water providing a minimum 10 Å distance between any atom of the solute and any edge of the box. Explicit K+ and Cl− ions were added at random positions at least 6 Å from solute atoms and 4 Å from each other to achieve 0.2 M excess KCl concentration with additional K+ ions to neutralize the system. The force field parameters were ff99SB (53) for the protein, parmBSC0 (54) for the DNA, TIP3P (55) for the water and TIP3P-specific ions (56). The complete system contained 61 042 atoms.

#### apo MTERF1

The procedure outlined above was repeated, except that the DNA was removed from the initial structure along with the crystallographic water. Initial simulations using an explicit solvent truncated octahedron with a 10 Å solvent buffer were found to be insufficient to enclose the protein during periods of large conformational change (data not shown). Thus, a minimum distance of 18 Å between the protein and any edge of the box was used, yielding a final dimension of 111.9 Å and 109.5°. Additional Cl− ions were added to neutralize the system, with 0.2 M excess K+ and Cl−. The same force field parameters were used. The complete system contained 98 124 atoms.

#### Search mode MTERF1–DNA complex

The procedure used for the specific MTERF1–DNA complex was used for the search mode MTERF1–DNA complexes, except the initial coordinates were taken from the poses generated by docking (see below). The coordinates of B-DNA were generated using NAB (57) and the 22 base pair cognate sequences.

#### Generating a non-specific complex

apo MTERF1 structures were considered sufficiently complementary to B-DNA (Supplementary Table S1) if superhelical pitch was less than 42 Å and radius between 9 Å and 16 Å (Supplementary Figure S3 and Section 4). The three lowest pitch structures of apo MTERF1 from each of the eight independent simulations were used for docking (see Supplementary Section 5 for docking details); three simulations produced no low pitch structures. The protocol was validated by docking MTERF1 and DNA from crystallography, reproducing the experimental complex (RMSD < 3 Å for the 7 highest ranked structures).

To distill the pool of potential non-specific complexes, the best ranked DOT poses were filtered by how well the protein tracked the major groove (Supplementary Section 6). In short, the major groove was geometrically defined as a sequence of sites (Supplementary Figure S4), and the average distance between all superhelical residues and their nearest major groove site (hereafter called the major groove distance) was calculated. We empirically chose a cutoff of 11 Å for the average distance—larger than the specific complex (7 Å, Supplementary Figure S5) but smaller than poses in which the binding cleft visually was not in contact with the major groove. A representative pose is shown in Supplementary Figure S6.

### MD equilibration and production

#### MTERF1–DNA specific complex

The multi-stage equilibration procedure is outlined in Supplementary Table S2. A 1 fs time step for dynamics and a 8.0 Å non-bonded direct space interaction cutoff were used, with PME (58) to calculate long-range electrostatic interactions across the periodic lattice containing the simulation cell. Initial minimization used the crystallographic structure as the reference, and subsequent stages used the final structure from the previous stage. Unless otherwise noted, the same force constant and ensemble were used in subsequent steps. Initially, all atoms added to the crystal structure were minimized while all atoms resolved by crystallography except crystallographic water (group A in Supplementary Table S2) were restrained with a force constant of 100.0 kcal/mol/Å^2^. The system was then heated in NVT from 100 K to 300 K linearly over 100 ps. Next, the density of the system was equilibrated at 300 K for 100 ps in NPT. With temperature and pressure equilibrated, MD continued at 300 K for 250 ps and the restraint force constant was decreased 10-fold. Since protein backbone atoms are often less susceptible to crystal packing forces, the restraint group was transitioned from all crystallographic heavy atoms to only the protein and DNA backbone atoms in the subsequent stages (group B in Supplementary Table S2). The system was minimized using a restraint force constant of 10.0 kcal/mol/Å^2^ and otherwise identical conditions as the initial minimization. Stage 6 was 100 ps of NPT dynamics at 300 K. The next two stages were identical to stage 6, except the restraint force constant was decreased to 1.0 kcal/mol/Å^2^ (stage 7) then 0.1 kcal/mol/Å^2^ (stage 8). The ninth stage of equilibration was again identical to stage 6, except positional restraints were completely removed. Stage 9 was 1.25 ns of unrestrained NPT. Thereafter, the NVT ensemble was used for unrestrained production. Independent trajectories involved simply initializing dynamics with velocities drawn from a Maxwell–Boltzmann distribution in stage 2.

#### apo MTERF1

Equilibrating apo MTERF1 followed the procedure above, except without the DNA present. Stage 9 was 2.25 ns of unrestrained NPT.

#### MTERF1–DNA search complex

The equilibration procedure above was used with only the following modifications. For each stage, one-tenth the restraint force constants were used because the non-specific complexes generated from docking were expected to be less precise than a high resolution crystal structure. Also, the DOT2.0 (59) energy function may have generated globally stable poses with locally unstable contacts that require flexible restraints to relax. The DNA restraint group was the same as the specific complex. In stages 1 through 4, only the Cα atoms of the superhelical residues were restrained. MTERF1 was not restrained during equilibration in stages 5 through 7. Stage 8, the final stage, was 250 ps of unrestrained NPT MD. Thereafter, production dynamics used the NVT ensemble. During production, each of the 12 search mode simulations switched to a 4 fs time step after ∼3 μs of MD, since the H-mass repartitioning algorithm in Amber became available (60).

## RESULTS

Exploring how TFs search DNA has been the focus of extensive experimental and theoretical research (61–63) yet many basic questions remain unanswered owing to the lack of structural data for unaltered non-specific protein–DNA complexes. MD simulations, both coarse grained (64,65) and atomistic (66,67), have been able to provide some of the needed structural insight into the transient states (∼μs) involved in search mode. Coarse-grained simulations lack atomistic resolution and internal flexibility to pinpoint specific interactions or DNA distortions that will be needed for a high-resolution mechanism of search and recognition, and previous atomistic MD simulation studies have relied on biasing potentials to generate search mode models.

### MTERF1 from crystallography clashes with B-DNA

**Figure 3.**
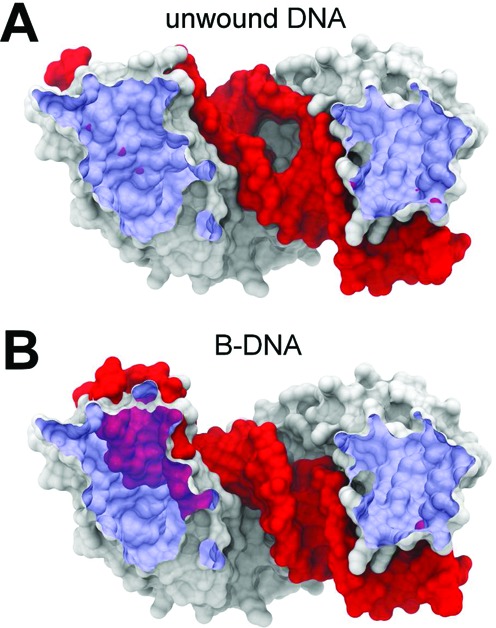
MTERF1 in the recognition mode conformation is too unwound to track the major groove of B-DNA. Surfaces were sliced to show incompatibility: the blue clipping plane appears purple where the DNA penetrates the protein. (**A**) Only minor steric clashes are present in the crystal structure of the recognition complex with unwound DNA. (**B**) Aligning the C-site P atoms of B-DNA to the corresponding region in the crystal led to large steric clashes between the N-site and the N-terminal domain of MTERF1. Alignment of the N-site resulted in similar clashes in the C-site.

As described above, we exclude models in which MTERF1 binds transiently predeformed DNA because its population would be much too low for efficient recognition. Thus, we tested the next simplest model, in which MTERF1 in the recognition conformation binds to B-DNA (Model A in Figure 2). Since the N- and C-sites of DNA in the crystal structure were essentially B-form (Supplementary Figure S1), we aligned the target cognate sequence in a B-form conformation to either site to generate potential search mode models. In contrast to MTERF1 and unwound DNA, large steric clashes occur between the molecular surfaces of MTERF1 and B-DNA (Figure 3A and B). Alternately, using a docking approach to find a more optimal threading of B-DNA through MTERF1, B-DNA passed through the binding cleft of the protein only when 10 steric clashes were permitted (Supplementary Figure S7). The structures were very high in energy and attempts to relieve the clashes using minimization and MD failed. Because the superhelical topology does not match that of B-DNA, the protein was unable to continuously track the major groove, suggesting that Model A may not be a reasonable paradigm for MTERF1 scanning DNA.

### Intrinsic axial and radial motions of MTERF1

**Figure 4.**
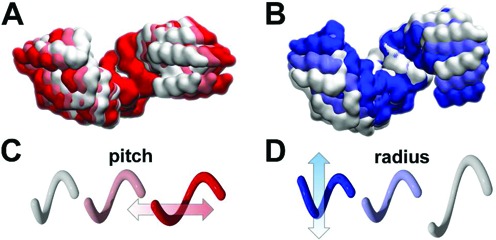
Lowest frequency modes of MTERF1 adapt superhelical pitch and radius and may permit binding to B-DNA. (**A**) The lowest frequency ANM mode of MTERF1 is an axial motion, white and red surfaces of Cα atoms denote positive and negative displacements, respectively. (**B**) The next lowest frequency ANM mode of MTERF1 is a radial motion, white and blue surfaces of Cα atoms are positive and negative displacements, respectively. (**C**) and (**D**) are cartoons of motions above, pitch and radius, respectively.

To track the major groove of B-DNA, MTERF1 must adopt an alternate conformation in search mode, corresponding to Model B in Figure 2. As the DNA helix in the recognition mode is unwound, we speculated that the MTERF1 superhelix in the recognition mode might also be unwound (higher pitch) relative to the search mode. Thus, we tested whether the MTERF1 topology possesses intrinsic motions that might lead to lower pitch conformations that better track a B-DNA major groove. To explore this hypothesis, we calculated the mechanical modes of the protein topology using an ANM. The results support the hypothesis. The global (lowest frequency) motions correspond to dynamics of the superhelical topology. To visualize superhelical motions, conformations were projected along the modes. Relative to the long axis of the protein, mode 1 is an axial motion and mode 2 is a radial motion (Figure 4). Importantly, dynamics along mode 1 may lead to a low pitch ensemble more compatible with tracking a B-DNA major groove.

ANM cannot determine the magnitude and the sense (positive or negative) of the displacement. However, the direction of ANM modes can overlap with those of atomistic MD (68), suggesting ANM and MD are complementary methods for characterizing protein dynamics. Similar motions predicted by methods with different limitations would suggest that the model for the dynamics is less likely to be an artefact. Furthermore, atomistic MD may give more detailed insight not only into the types of dynamics encoded in the MTERF1 topology, but also quantify the ranges of pitch and radius that are sampled at ambient temperature, and whether these are compatible with binding a B-DNA duplex.

To establish a baseline for analysing the apo MTERF1 dynamics, we performed 4 independent 1.5 μs control simulations of the MTERF1–DNA specific complex. We expected small structural fluctuations around an average conformation similar to the crystal structure. To quantify similarity, we measured the root mean square positional deviations (RMSD) between our MD snapshots and the equilibrated crystal structure using cpptraj (69). The evolution of RMSD in the control simulations (Supplementary Figure S8A, B) shows that the conformation of MTERF1 throughout the simulations of the specific complex remains similar to that of the reference.

We next generated an ensemble of apo MTERF1 structures by performing eight independent 0.3 μs MD simulations. In contrast to the control simulations of the specific complex, RMSD analysis on the apo MTERF1 simulations indicate that, in the absence of DNA, the protein undergoes significant conformational change with respect to the same reference (Supplementary Figure S8C). To gain further insight into the nature of these changes in the apo protein structure, we compared the motions sampled in MD to the global modes obtained from the ANM calculations.

### MD and ANM exhibit similar low frequency motions

To measure the similarity of ANM and MD motions, we used principal component analysis (PCA) on the complete MD ensemble to obtain the top ten principal components (PCs) of apo MTERF1 conformational fluctuations. The 10 lowest frequency ANM and MD vectors show high similarity as indicated by an RMSIP (50) of 0.77 (Supplementary Table S3). The similarity indicates that the global dynamics sampled in the atomistic MD simulations also correspond to changes in the superhelical pitch and radius of MTERF1, as was suggested by ANM (Figure 4). Observation of similar dynamics in the two different computational approaches also suggests that the results are less likely to be an artefact of a specific model. We next analysed the range of fluctuations in these measures to determine whether these dynamics could result in structural excursions that would remodel the apo MTERF1 binding site to accommodate B-DNA without steric clashes.

### Quantifying MTERF1 superhelical motions using a general gauge of helical parameters

We hypothesized above that compatibility of MTERF1 and B-DNA would encompass similarity in the global helical pitch. The challenge is that no gauge of global helical pitch and radius exists for proteins, while DNA is naturally defined by helical coordinates. The pitch of DNA depends on the rise between each base pair step—the displacement along the helical axis—and the twist of the step—the rotation of the base pair plane about the helical axis. These parameters depend on a well-defined helical frame, which is well established for DNA (70) but has not been described for proteins. We thus implemented an approach of defining a helical frame to quantify MTERF1–DNA complementarity. The helical topology of MTERF1 arises from its modular architecture and we cast our new helical reference frame with the assumption that a helical axis exists for MTERF1 and, importantly, that the axis is normal to the plane onto which the protein projects the best circle (see Methods and Supplementary Section 2). We identified a set of proline residues (with two exceptions, see Methods) that occupy comparable positions within each motif, referred to as the superhelical residues (Figure 1A). As the superhelical residues track the major groove, the radius of the resulting helix defined with these residues is expected to closely match that of the bound DNA. We elaborate on the method in Section 2 and its application in Sections 3 and 9 of the Supplement.

### apo MTERF1 spontaneously adopts structures with the same pitch as an average B-DNA

**Figure 5.**
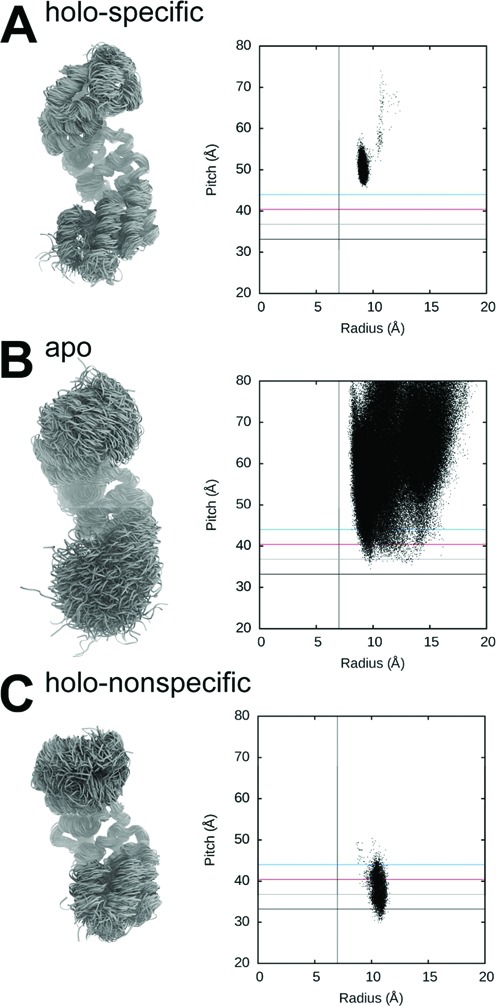
A switch in the MTERF1 superhelical topology. (**A**) In recognition mode, unbiased MD simulations show that MTERF1 populates a high pitch state consistent being bound to unwound (high pitch) DNA. The DNA is not shown for clarity. To show the expected range of B-DNA pitch, horizontal lines mark the average structure of B-DNA (black) plus one, two and three standard deviations (grey, red, blue, respectively). A vertical guide is placed at 7 Å to represent B-DNA radius compatibility. (**B**) In the absence of DNA, apo MTERF1 samples a wide range of superhelical conformations, extending into the range compatible with B-DNA. (**C**) In search mode, the superhelical dynamics of MTERF1 are suppressed by B-DNA with a much narrower distribution of both pitch and radius. Compared with holo-specific, the small increase in radius of holo-nonspecific is likely caused by the decrease in pitch. Snapshots of MTERF1 were selected evenly from concatenated trajectories of the respective ensembles; the N-terminus is towards the top.

We carried out helical analysis on the MD simulations for apo and holo (specific complex) MTERF1. The superhelical dynamics of apo MTERF1 are strikingly different from holo MTERF1, with the ensemble sampling a much broader range of pitch and radius for the apo protein (helical parameters along with representative structures are shown in Figure 5A and B). Comparing the standard deviations of the superhelical parameters for the two ensembles indicates that apo MTERF1 superhelical radius and pitch are roughly one order of magnitude more diverse than holo-specific (Supplementary Figure S9). Interestingly, the broad range of superhelical radius values sampled by the apo protein has a lower bound of ∼7 Å, the radius of a B-DNA major groove (Supplementary Table S1), suggesting that although the type of motion is encoded in the topology, the protein may lack selective pressure to increase flexibility beyond that required for function. The ensemble sampled by the apo MTERF1 simulations also exhibits structures with superhelical pitch similar to that of B-DNA (Figure 5B) while, as expected, MTERF1 in the control simulation remains much higher than B-DNA (Figure 5A).

### Are low pitch apo MTERF1 structures compatible with a B-DNA major groove?

Although the helical analysis suggests that apo MTERF1 spontaneously adopts structures with superhelical pitch and radius compatible with B-DNA, these global measures of structure cannot confirm that the structure complementarity is sufficient to avoid the steric clashes that were obtained when the crystal conformation was docked to B-DNA (Figure 3). We therefore repeated the docking procedure using low pitch apo MTERF1 structures along with canonical B-DNA, and subsequently performed MD to relax the docked complexes and determine if they provide reasonable and stable models of the non-specific complex. As a control, we also separated and then re-docked the DNA and protein structures from the crystal structure of the recognition complex; this control successfully recapitulated the crystal structure and MD simulations of the resulting complexes were stable. We thus proceeded with docking the low pitch apo structures to B-DNA.

#### Docking and scoring low pitch apo MTERF1 and B-DNA

To obtain a diverse set of docking poses mimicking productive non-specific complexes, we docked to B-DNA the lowest-pitch protein structures from the five apo MTERF1 simulations that sampled conformations with superhelical pitch <42 Å. This pitch cutoff was selected since it represents a statistically significant population of B-DNA structures (11,15,71) and thus is likely compatible with the major groove of B-DNA (Supplementary Section 4). Apo structures in this range also have radii larger than 9 Å (Figure 5B), suggesting that inward facing sidechains should fit over the major groove of B-DNA (5.7 Å Supplementary Table S1).

We independently docked 14 low pitch apo MTERF1 structures to B-DNA. In each of the 14 calculations, the energy of 54 000 poses was evaluated using the DOT2.0 energy function (see Materials and Methods) and only the 30 lowest energy poses were retained. Next, productive poses in which MTERF1 tracked the major groove were filtered from poses that did not by measuring the distance between superhelical residues and major groove sites (see Materials and Methods and Supplementary Section 6). We considered acceptable values to range from a lower limit of ∼7 Å (obtained from the specific complex Supplementary Figure S5) up to 11 Å, beyond which poses did not visually appear to closely track the major groove (see Supplementary Figure S6 for examples). Thirteen poses fell within this range, and after culling one due to a steric clash, 12 poses were retained for further analysis (Supplementary Figure S10).

#### Relaxing the docked poses using MD

To optimize and relax the docked complexes, and establish the stability of the search mode model, we simulated the productive poses using MD. The 12 docked poses were equilibrated and subjected to 3 μs of unrestrained MD. The complementarity of the protein–DNA interface increased during the simulations, as measured by shared surface area (Supplementary Figure S11). Consistent with a weak binding model of search mode, the shared surface areas were less than that measured during MD of the specific complex (Supplementary Figure S12). Despite looser binding, MTERF1 remained in contact with the major groove, indicated by stable time courses for the protein-major groove distance (Supplementary Figure S11). The superhelical pitch and radius of MTERF1 from a representative simulation (Figure 5C) samples low pitch and radius metastates, the distributions of which are much narrower than apo MTERF1 (Figure 5B). This suggests the protein occupies a metastable conformational state complementary to B-DNA in the non-specific complex. Overall, the observation of stable docked complexes with increased complementarity supports our hypothesis that the extensive superhelical dynamics of apo MTERF1 allow it to sample low pitch structures that are compatible with binding to a B-form DNA duplex.

**Figure 6.**
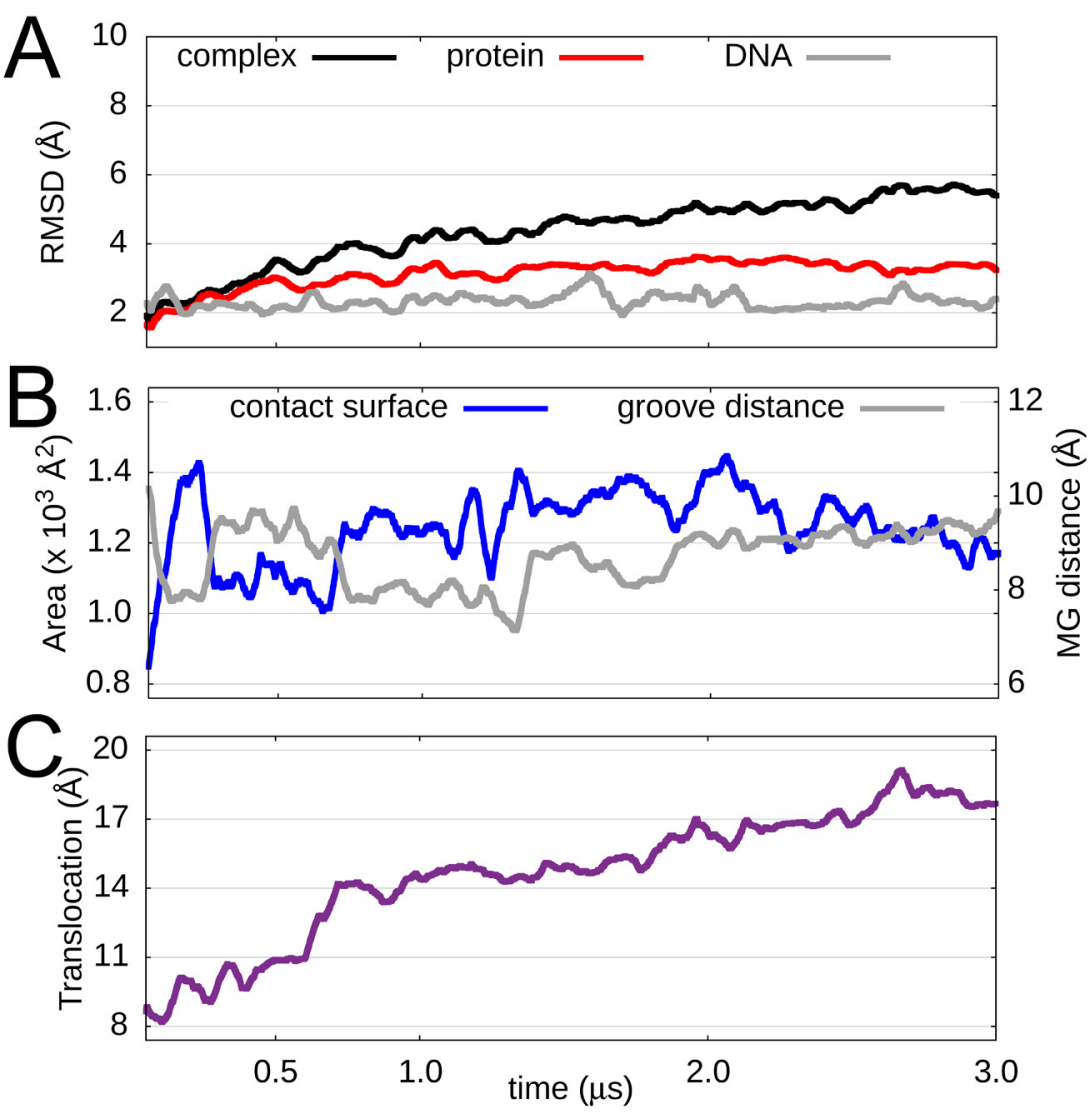
MTERF1 in search mode. (**A**) Structural stability of the search mode complex as measured by the backbone RMSD of the DNA (grey), MTERF1 (red) and the MTERF1-DNA complex (black), the last two of which were aligned to the protein; all used the structure at 20 ns as the reference, to account for docked pose relaxation. While the protein RMSD remains stable, that of the complex steadily rises. (**B**) Time dependence of the contact surface area shared by MTERF1 and the DNA (blue) and the major groove distance (grey). (**C**) Distance between the centres-of-mass of protein and DNA; increasing values with time suggest change in the location of the protein on the DNA. Data shown are averaged with a 50 ns sliding window.

To gain more insight into the conformational changes that accompany the increasing surface complementarity of MTERF1 and DNA, we evaluated the RMSD of the protein, the DNA and the complex for a representative simulation (Figure 6A) along with the interface analysis discussed above (Figure 6B). Using a reference snapshot taken after 20 ns of MD (to account for initial relaxation), the DNA and protein structures were stable with an RMSD remaining near 2 Å and 3 Å, respectively, during the entire MD run. This suggests that the increased structural complementarity involved relatively small changes to the protein and DNA structure, confirming our hypothesis that low pitch MTERF1 structures could accommodate B-DNA.

Calculation of the RMSD for the entire complex resulted in relatively low values during the first microsecond of the simulation, consistent with the argument that the docked poses were stable following modest relaxation. For the final 2 μs, however, the RMSD of the complex drifts to higher values, eventually reaching 5 Å. The stable contact surface area suggests that the high RMSD value does not correspond to dissociation of the docked complex. We therefore investigated the possibility that the high RMSD might arise from functionally relevant dynamics of MTERF1 in search mode.

### MTERF1 undergoes 1D sliding during microsecond MD

**Figure 7.**
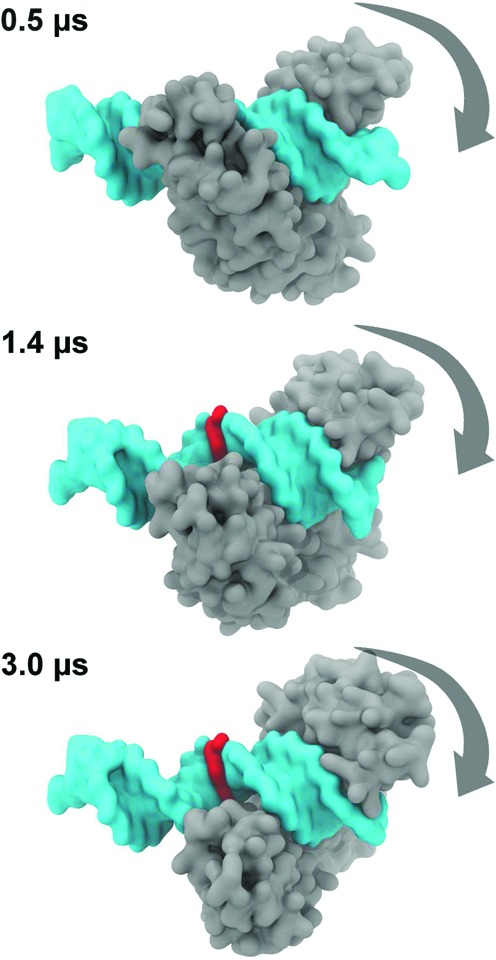
Snapshots of the complex at different time points, with MTERF shown in grey and DNA in aqua with the central bp coloured red to visually highlight MTERF1 translocation along the major groove of B-DNA. Snapshots are RMS aligned to only the DNA backbone so that MTERF1 is seen to move (rightward) with respect to the DNA frame (indicated by arrows).

Single-molecule fluorescence of eight different proteins sliding on DNA (72) suggests an accurate model of MTERF1 in search mode might exhibit spontaneous sliding along the major groove on the μs-timescale. To measure sliding, we define the translocation distance as the distance between the centre of mass (COM) of the superhelical residues and the COM of the DNA (Figure 6C and Supplementary Figure S13); this approximates the location of MTERF1 on the DNA since the contact surface indicates that the protein remains in the major groove. As shown in Figure 6C, this distance increases with time from an initial value of 8 Å to 17 Å, corresponding to sliding of 3 bp since the rise along each bp step is ∼3 Å. During the first 0.5 μs, the translocation distance changes from an initial value of 8 Å to 11 Å, which may also correspond to initial relaxation of the docked protein into the major groove. After 1 μs, this distance increases again, to 14 Å; after the second microsecond, the distance becomes 17 Å, suggesting an approximate sliding rate of ∼1 bp/μs that is consistent with experimental measurements on other protein–DNA complexes (72). After 3 μs, the protein reaches the end of the duplex that was used for the docking simulations. To visually confirm sliding, we examined snapshots of the search mode complex that were fit to the DNA (Figure 7). In these complexes, MTERF1 can be seen to diffuse along the DNA in the major groove. We conclude that the docking of low pitch apo MTERF1 to B-DNA leads to a dynamic model of MTERF1 in search mode.

### A model of the MTERF1–DNA search and recognition mechanism

**Figure 8.**
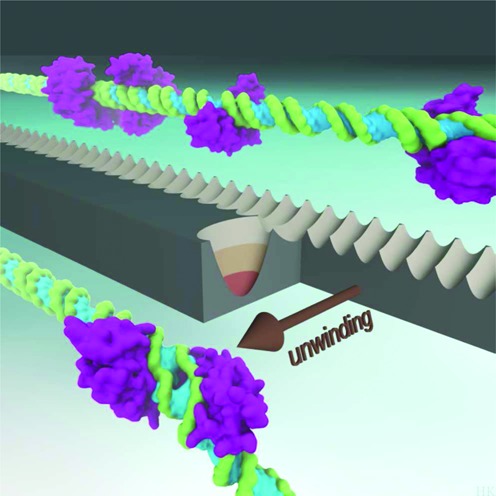
A model of the MTERF1 search and recognition landscape based on protein intrinsic superhelical motions. Helical motions drive translocation, and presumably for all but the target sequence, these motions are modulated so that the protein cannot fully unwind DNA before sliding on to the next site. At the target, the height of the unwinding barrier is sufficiently low for the protein to switch into recognition mode and unwind DNA rather than sliding to the next site.

The simulations described here provide a model of the dynamic MTERF1 non-specific complex, supporting a hypothesis for the search and recognition mechanism. Based on our observation that the K-rich C-tail of MTERF1 is often unstructured, condensation of the protein onto DNA may be driven by a fly-casting mechanism (39). It is possible that MTERF1 may follow a hybrid conformational selection-induced fit mechanism (73), in which a more unstructured MTERF1 folds in the proximity of the DNA. As MTERF1 collides with DNA, the intrinsic protein motions open the binding cleft to permit productive binding of B-DNA in a manner consistent with gated binding (40). Once productively bound—the search mode described above—the MTERF1 superhelix is confined to helical motions compatible with the low pitch and low amplitude helical motions of B-DNA (Figure 5C). The precise motions and the degree to which they are coupled likely depend on sequence (15,17), indirect readout, and shape readout (74,75). Generally, small barriers to sliding separate fleeting non-specific complexes whose energy gaps are small (Figure 7). The intrinsic superhelical motions (Figure 4) not only allow the apo protein to bind DNA, but may also be a crucial factor in the ability of MTERF1 to unwind the target DNA, in which sequence and structure-dependent polarization may be key (76,77). Contact with the target sequence switches the protein into recognition mode, accompanied by a conformational switch from low pitch (Figure 5C) to high pitch (Figure 5A) that drives DNA unwinding and base-flipping (Figure 1), likely with energetic compensation between DNA strain and formation of specific recognition contacts. The conformational switch is likely fast to allow efficient search and recognition, but the energy gaps are enlarged (78), leading to tight binding and a kinetic roadblock mechanism. A cartoon of the putative search and recognition landscape is shown in Figure 8. Thus, we propose MTERF1 follows an allosterically modulated gated search and recognition mechanism in which the amplitude of the superhelical pitching motion is attenuated by direct and indirect readout. Future work will build on the model presented here to explore subsequent steps in specific recognition, with possible implications for genome editing reagent design (79,80).

## CONCLUSION

We proposed several potential models for the non-specific complex of transcription factors bound to B-DNA, using MTERF1 as a model TF. Our analysis indicated that conformational change of the TF was required, and MD simulations provided a model for the dynamic ensemble of apo MTERF1 structures. Analysis of the intrinsic motions indicated that dynamics of the superhelical topology characterize the changes during binding, and perhaps also during search and sequence recognition. Docked complexes provided reasonable models for the non-specific complex, as indicated by low RMSD values, high surface area complementarity, and, on longer timescales, 1D diffusion (sliding) of the protein along the DNA major groove. The resulting dynamic model for this transcription factor carrying out non-specific binding and search provides a view of protein–DNA recognition complementary to that obtained from a wealth of crystal structures of stable recognition complexes.

## Supplementary Material

SUPPLEMENTARY DATA
