# Supplementary material for: A human transcription factor in search mode
Source: Nucleic Acids Res. 2015 Dec 15;44(1):63–74. doi: 10.1093/nar/gkv1091 (PMC4705650; doi:10.1093/nar/gkv1091)
Supplement: SUPPLEMENTARY DATA [file supp_44_1_63__index.html]

A human transcription factor in search mode — SUPPLEMENTARY DATA 

# A human transcription factor in search mode

## SUPPLEMENTARY DATA

- SUPPLEMENTARY DATA
- SUPPLEMENTARY DATA
