# Supplementary material for: A human transcription factor in search mode
Source: Nucleic Acids Res. 2015 Dec 15;44(1):63–74. doi: 10.1093/nar/gkv1091 (PMC4705650; doi:10.1093/nar/gkv1091)
Supplement: SUPPLEMENTARY DATA [file supp_gkv1091_a_human_TF_in_search_mode---KSuppl--2015-11-25.pdf]

A human transcription factor in search mode

Kevin Hauser[1,2], Bernard Essuman[3], Yiqing He[4], Evangelos Coutsias[1,5], Miguel Garcia-Diaz[6], and Carlos Simmerling[1,2,*]

[1] Laufer Center for Physical and Quantitative Biology, Stony Brook University, Stony Brook, New York, 11794, United States of America

[2] Department of Chemistry, Stony Brook University, Stony Brook, New York, 11794, United States of America

[3] Suffolk Community College, Selden, New York, United States of America

[4] Great Neck South High School, Great Neck, New York, United States of America

[5] Department of Applied Mathematics and Statistics, Stony Brook University, Stony Brook, New York, 11794, United States of America

[6] Department of Pharmacological Sciences, Stony Brook University, Stony Brook, New York, 11794, United States of America

* To whom correspondence should be addressed. Tel: 1-631-632-5324; Email: carlos.simmerling@stonybrook.edu

SUPPLEMENTARY INFORMATION

Contents

Section 1. DNA helicoidal analysis of the MTERF1 recognition complex 2

Section 2. Helix fitting 2

Section 3. Anisotropic Network Model details 6

Section 4. Finding DNA parameters with our general helix frame 6

Section 5. Generating a nonspecific complex 7

Section 6. Calculating how well MTERF1 tracks a major groove 7

Section 7. Equilibration details 12

Section 8. Docking B-DNA to MTERF1 from the recognition structure 12

Section 9. RMSD analysis of control and apoMTERF1 simulations 13

Section 10. The similarity of ANM and MD lowest frequency motions 15

Section 11. Histograms of holo MTERF1 and apo MTERF1 helical parameters 16

Section 12. Productive, energetically stable nonspecific docked complexes 17

Section 13. Quantifying how tightly MTERF1 binds DNA 18

Section 14. Measuring MTERF1 translocation along DNA 20

REFERENCES 20

SECTION 1. DNA HELICOIDAL ANALYSIS OF THE MTERF1 RECOGNITION COMPLEX

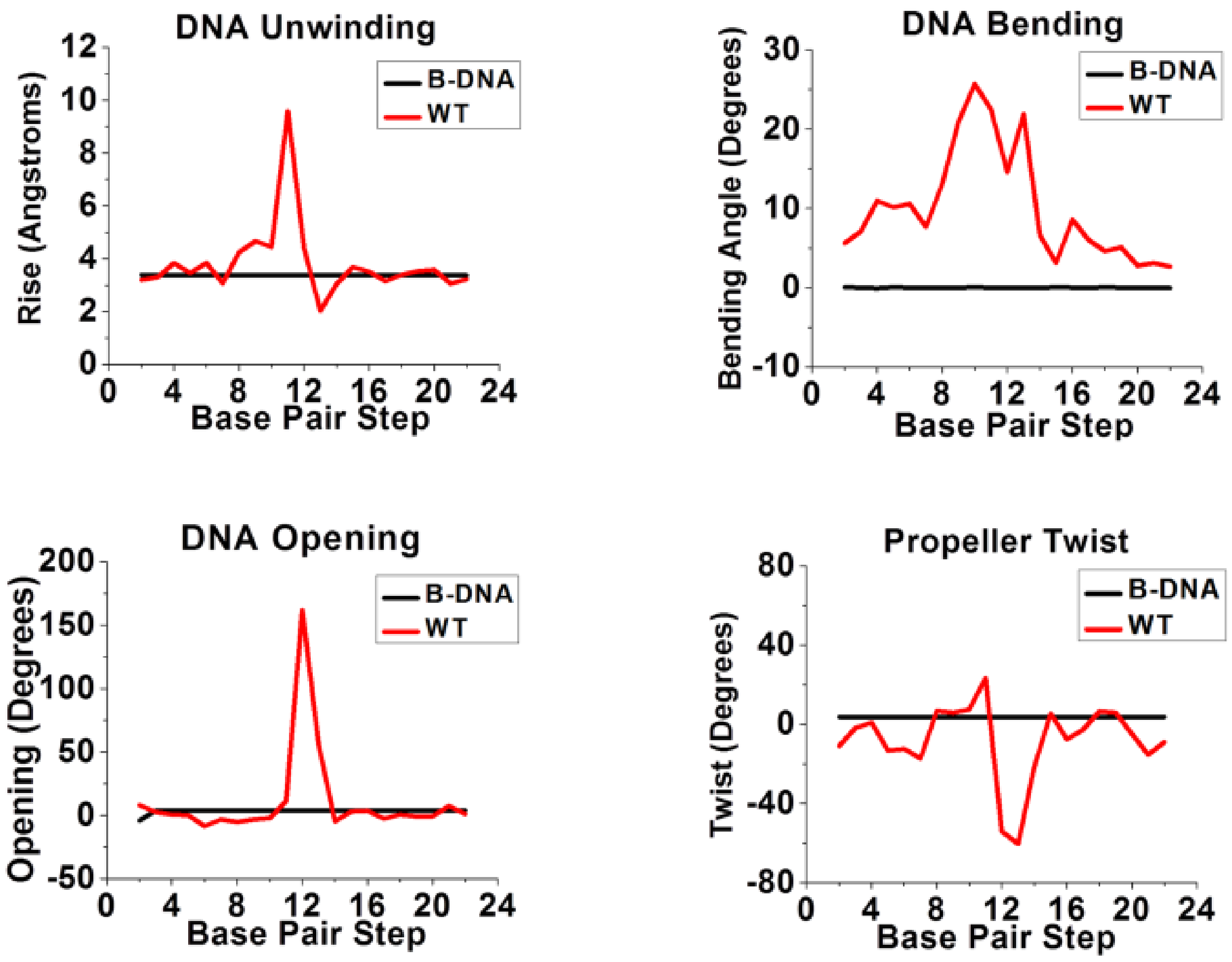

Figure S1. Structural analysis of DNA in the MTERF1-DNA specific complex (1). Using Curves (2), the base pair step parameters rise distance (top left), bending angle (top right), opening angle (bottom left), and twist angle (bottom right) were calculated. For reference, the parameters for B-DNA are shown in each panel.

SECTION 2. HELIX FITTING

The goal of the helical analysis was to characterize the dynamics of MTERF1 by detecting whether its helical geometry had adapted to the helical curve of a B-DNA groove. The challenge was two-fold: MTERF1 did not present a helix of points spaced regularly; there were only nine points to work with.

An ideal circular helix threads a cylindrical surface. The length of the line connecting two successive points of intersection between the generator of the cylinder and the helix defines helical lift. Intrinsic helical pitch is the ratio of the lift and the circumference of the basic cylinder. The pitch for a sequence of regularly spaced points along a helix can be defined by a regular polygon line (3). Helical parameters can be readily obtained for molecular systems with regularly spaced atoms that are symmetric about the helical axis (4). For MTERF1, recurring proline residues that tracked the major groove of unwound DNA were identified (**Figure 1.A**). However, the proline residues are not regularly spaced and thus the above method of Cochran is not appropriate for MTERF1. In general, few methods can parameterize helices that make one or fewer convolutions such as the helix of MTERF1 (5).

We required a more general method for points that are not regularly spaced along a helix with less than one convolution. The general, total least squares approach of Nievergelt (6) can fit such a helix by seeking the best-fit right cylinder that is wrought by an irregular helix. We implemented a similar but more elementary approach based on the trivial property that a right cylinder projects a circle on the plane whose normal is parallel to the longitudinal axis of the cylinder (7).

The method used here seeks the plane onto which the postulated helix projects the best possible circle. The plane is numerically optimized by minimizing the residual of the fit to a circle as a function of the orientation of the projection plane. The optimization occurs in two steps: (1) Rotate the plane using an orthogonal transformation and project the postulated helix onto it; (2) Fit the projection to a circle by solving a linear least squares problem with singular value analysis.

The plane is defined in terms of the direction cosines (a,b,c) = (sinφcosθ, sinφsinθ, cosφ) with φ and θ the spherical coordinates. The nine Cα of the MTERF1 superhelical residues were rotated from their original Cartesian coordinates (*X,Y,Z*) to new coordinates (*x,y,z*),

$$\begin{pmatrix} x \\ y \\ z \end{pmatrix} = \begin{pmatrix} \sqrt{1-a^2} & 0 & a \\ -ab/r & c/r & b \\ -ac/r & -b/r & c \end{pmatrix} \begin{pmatrix} X \\ Y \\ Z \end{pmatrix} \qquad \textbf{Eqn. S1}$$

where the r of transformation is $\sqrt{b^2+c^2}$. The projection depends only on the plane's orientation, and not on its position relative to the origin because the orthogonal transformation conserves the internal helix structure; the plane is assumed to pass through the (arbitrary) origin. On the projection plane, the centre and radius of the circle are determined from the linear least squares problem,

$$\begin{pmatrix} 1 & 2x_1 & 2y_1 \\ 1 & \dots & \dots \\ 1 & 2x_N & 2y_N \end{pmatrix} \begin{pmatrix} k \\ x_0 \\ y_0 \end{pmatrix} = \begin{pmatrix} x_1^2+y_1^2 \\ \dots \\ x_N^2+y_N^2 \end{pmatrix} \qquad \textbf{Eqn. S2}$$

with ρ the radius of the helix and (x_0,y_0) its centre, *k* is ρ^2-(x_0^2+y_0^2). The orientation of the plane, φ and θ, are optimized numerically by minimizing the square of the circle-fitting residual,

$$Res^2 = \sum_{i=1}^{N}(x_i - x_0)^2 + (y_i - y_0)^2 + \rho^2 - 2\rho\sqrt{(x_i - x_0)^2 + (y_i - y_0)^2} \quad \textbf{Eqn. S3}$$

Pitch is $|z_N\text{-}z_1|\Phi/2\pi$, where Φ is the parametric angle of the circle (helical sweep) and $|z_N\text{-}z_1|$ is the lift.

Fitting the helical parameters of apo MTERF1 using constraints

MTERF1 conformations with the lowest fit residuals (the best formed helices) were those with helical parameters most similar to B-DNA (the most important conformations in this work). Because we also observed pathological geometries due to collinearities, these geometrically interesting collinearities were analyzed. First, the full range of projection plane orientations were analyzed for a representative apo MTERF1 simulation (**Figure S2.A**). A heat-map of the fit-residuals as a function of the projection plane orientation (**Figure S2.B**) shows a correlated change in φ/θ that should affect the intrinsic pitch of the helix. Heat-maps of the radius (**Figure S2.C**) and pitch (**Figure S2.D**) reveal how the parameters change as expected when optimized to different projection planes as MTERF1 adopts extended conformations. The well formed conformations are shown in **Figure S2.E,F** and a pathological extended conformation is shown in **Figure S2.G**. To treat the pathology of extended conformations, constraints on the orientation of the projection plane were prescribed by limiting the range over which the planes could be rotated to that of the putative helix-type one, φ in [50°,70°] and θ in [240°,300°], the region containing the lowest residuals.

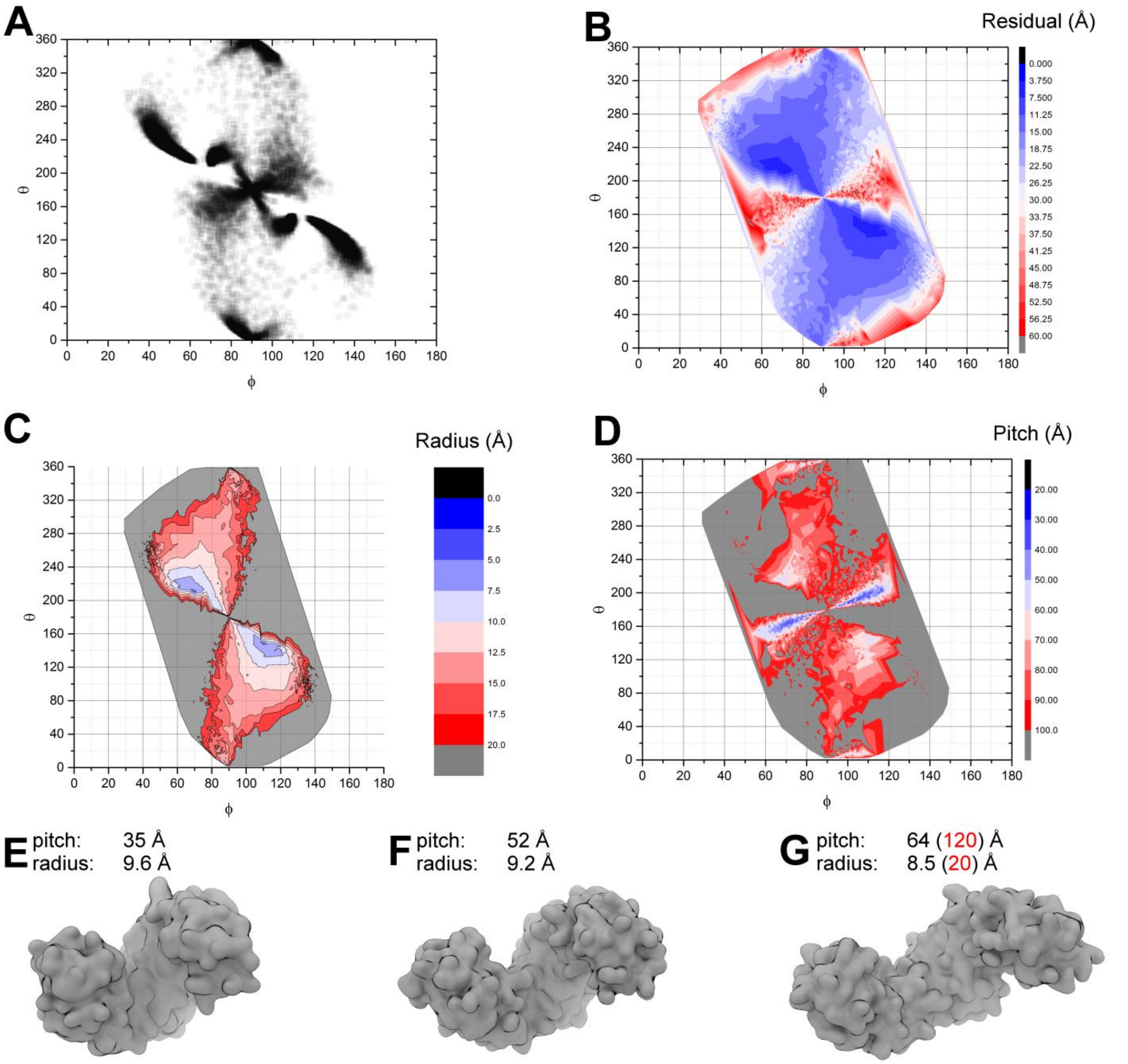

Figure S2. Calculating superhelical parameters of apo MTERF1. (**A**) Scatter plot of superhelical axes. Heat map of (**B**) residual, (**C**) radius, and (**D**) pitch. (**E**) Low pitch apo MTERF1. (**F**) High pitch apo MTERF1, the conformation corresponding to the structure found in the specific complex. (**G**) Very high pitch apo MTERF1. In (**E**) and (**F**) the unconstrained helical axis orientations were in φ in [50°,70°] and θ in [240°,300°] whereas the superhelical residues of the structure in (**G**) adopted an orientation in an alternate region of the map, the pitch and radius of which are shown in red. Data represents one of the 8 apo MTERF1 simulations.

SECTION 3. ANISOTROPIC NETWORK MODEL DETAILS

A Hessian matrix was constructed and diagonalized to calculate the normal modes. A distance cutoff that provided high correlation between calculated and experimental B-factors (8) was chosen. Cutoffs of 8, 10, 12, 15, 18, 21, and 24 Å were tested (8) resulting in correlation coefficients of 0.5778, 0.6209, 0.6217, 0.6229, 0.6311, 0.6472, and 0.6619, respectively.

SECTION 4. FINDING DNA PARAMETERS WITH OUR GENERAL HELIX FRAME

An ideal DNA geometry with a base pair step rise of 3.38 Å and a step twist of 36.0 degrees was analysed (**Table S1**). As expected, our method reproduces the helical rise parameters of B-DNA built using NAB (9) (36° twist, 10 base pair per revolution multiplied by 3.38 Å = 33.8 Å). Our method also reproduces twist. The radius of the major groove was defined as the radius of the helix traced by centre of the C1' atoms.

Table S1. Summary of helical parameters calculated by our method for ideal B-DNA.

Atoms	Count	Residual (Å)	Radius (Å)	Pitch (Å)	Rise (Å)	Twist (°)
**C1' (W)**	22	0.023	5.85	33.80	3.38	36.00
**C1' (C)**	22	0.015	5.85	33.81	3.38	36.00

(W) indicates the Watson strand, (C) the Crick strand in the ABC definition (10). The residual in our fitting procedure measures the deviation of the projected atomic coordinates from a perfect circle in units of Å (**Section 2**). Our sweep parameter is analogous to twist.

What is the pitch of B-DNA?

An upper limit of 42 Å B-DNA pitch was identified to be that which MTERF1 could bind in search mode. This value was arrived at by using two approaches. First, the literature in which the base pair step parameters rise was reported was reviewed (rise was multiplied by 10 since 10 bp/360°). Second, the helical pitch of B-DNA of our own MD simulations was measured.

A maximum value of rise in the central dinucleotide of CGCA/TGCG is 4.5 Å in nucleosome core particle crystal structures (11), which is almost identical to the average rise for all DNA sequences plus 3 standard deviations (4.4 Å) found from MD simulations of the 136 tetranucleotide sequences (12). Pitch for B-DNA, which contains 10 nucleotides per helical turn, is then 44 Å (**horizontal lines in Figure 5**). In a third example from the literature, Olson et al. reported average rise values of 3.36 Å and standard deviations of 0.25 Å of protein-DNA complexes in which complexes containing broken base pairs were omitted (41.1 Å pitch for B-DNA) (13). Together, the literature supports the assumptions that a B-DNA molecule a protein would randomly encounter in solution would likely posses a ~42 Å pitch, or less.

4 independent MD simulations of B-DNA were then performed. The systems were built, equilibrated, and production performed exactly as the search mode complexes. The helical parameters of the B-DNA in the MD trajectories were then analysed (**Figure S3A and S3B**). Overall, the distributions of DNA pitch agree with the above conclusions. The average pitch was observed to be 35.8 Å for the HS and 33.3 Å for the LS, with 2.31 Å standard deviation in the HS and 3.35 Å standard deviation in the LS. Thus the HS accesses pitch with 42.7 Å pitch (average plus 3σ) and the LS, 43.4 Å.

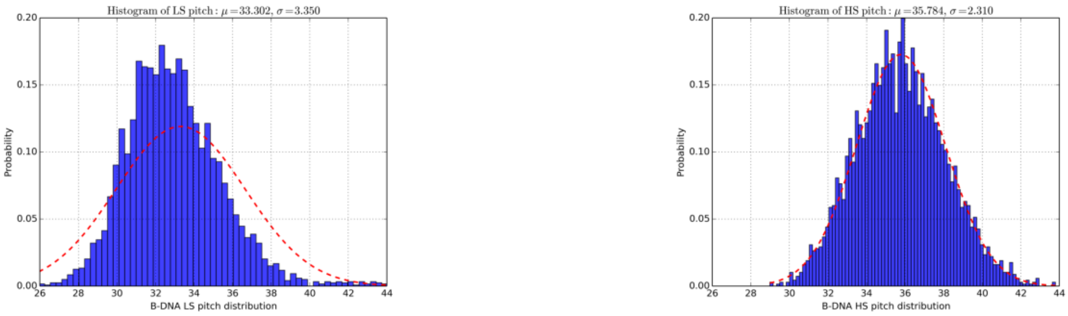

Figure S3. Control simulations of the 22 bp target sequence in a B-DNA geometry. (**A**) DNA light strand (LS) and (**B**) heavy strand (HS). Four independent 1 µs MD simulations were performed. Histogram used 100 bins.

SECTION 5. GENERATING A NONSPECIFIC COMPLEX

Thus 15 apo MTERF1 structures were docked to B-DNA using DOT2.0 (14), which has been previously been shown to be suitable for protein-DNA docking (15). Our procedure followed previously published protocols (14). Briefly, REDUCE (16) parameters for heavy and polar hydrogen protein and DNA atoms were used, electrostatic potentials were calculated with APBS (17) and 0.200 M ionic strength, and electrostatic clamping was used to flatten pathological energies (14). van der Waals energies were estimated by counting DNA atoms that were within an interaction region coating the protein; the inner surface of the region was defined by the MSMS (18) molecular surface with a 1.4 Å probe radius, and the outer surface was defined by expanding the protein van der Waals atomic radii by 3.0 Å (14). Desolvation energies were not included in the calculations. 54,000 orientations of apo MTERF1 and B-DNA were evaluated for each of the low pitch protein structures. The protocol was validated by docking MTERF1 and DNA from crystallography, reproducing the experimental complex (RMSD < 3 Å for the 7 highest ranked structures).

SECTION 6. CALCULATING HOW WELL MTERF1 TRACKS A MAJOR GROOVE

To test whether the highest ranked (lowest-energy) docked poses were reasonable models of a nonspecific complex, the 30 best poses from each of the 15 docking calculations were filtered by how well MTERF1 tracked the major groove, with the correct polarity. First, major groove sites were defined (**Figure S4A**). For B-DNA, a line connecting P atoms on opposite strands separated by 5 nucleotides (the P of nucleotide 1 on one strand and the

P of nucleotide 6 on the other strand) is nearly parallel to the helical axis with a length of 21 Å. The midpoint of the line, ~10 Å from the P atoms and ~8 Å from nucleobase functional groups, was defined as a major groove site (**Figure S4B**). The distance between superhelical Cα atoms and major groove sites was measured, and each were expected to be a distance of 6-10 Å from each major groove site, based on inspection of the N- and C-site in the crystal structure and analysis of simulations with the specific MTERF1-DNA complex (~7 Å, see **Figure S5**). Poses were classified as nonspecific complexes when MTERF1 tracked the major groove and consecutive superhelical Cα atoms resided in consecutive major groove sites. An average distance between these protein residue and major groove site pairs was < 11 Å when the docked pose visually appeared to be tracking the major groove (**Figure S6**). The pairing sequence of Cα superhelical atoms and major groove sites was set by the pair with the smallest separation for a particular pose. For example, if the smallest separation between a superhelical Cα and a major groove site was the fourth Cα and the fifth major groove site (a pair), then the first Cα was automatically paired with the second major groove site, the second Cα paired with the third major groove site, and so on. The pairing rule causes very large Cα-major groove site distances (>25 Å average) when MTERF1 is docked in the reverse polarity, or criss-crosses the major and minor grooves (the screws are cross-threaded).

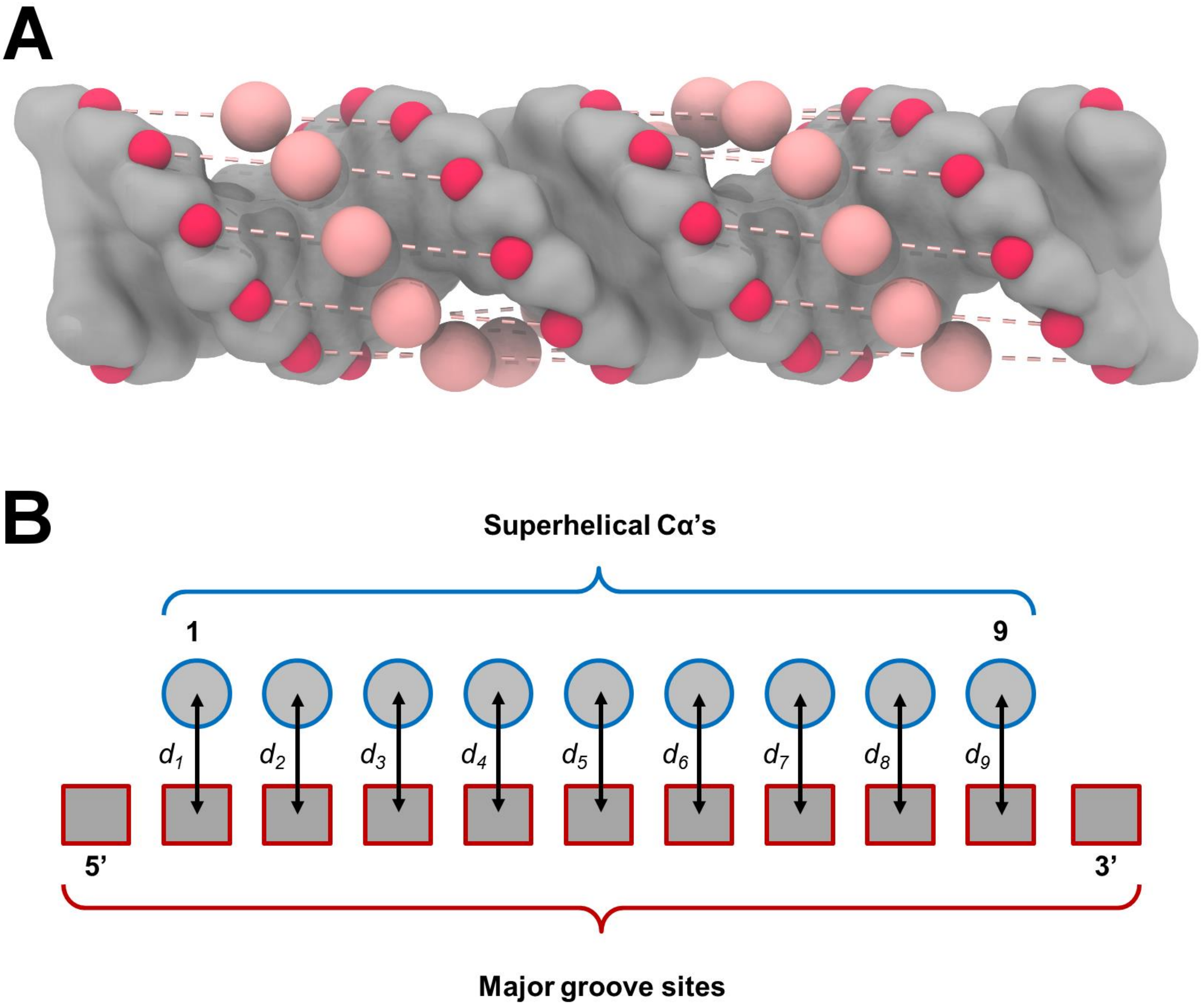

Figure S4. Method to geometrically measure how well MTERF1 tracks a major groove. (**A**) Major groove sites are the midpoints(pink spheres) between successive P atoms (dark red spheres) on opposite strands offset in sequence by 5, shown as pink dotted lines. (**B**) Major groove site-superhelical residue pairing scheme.

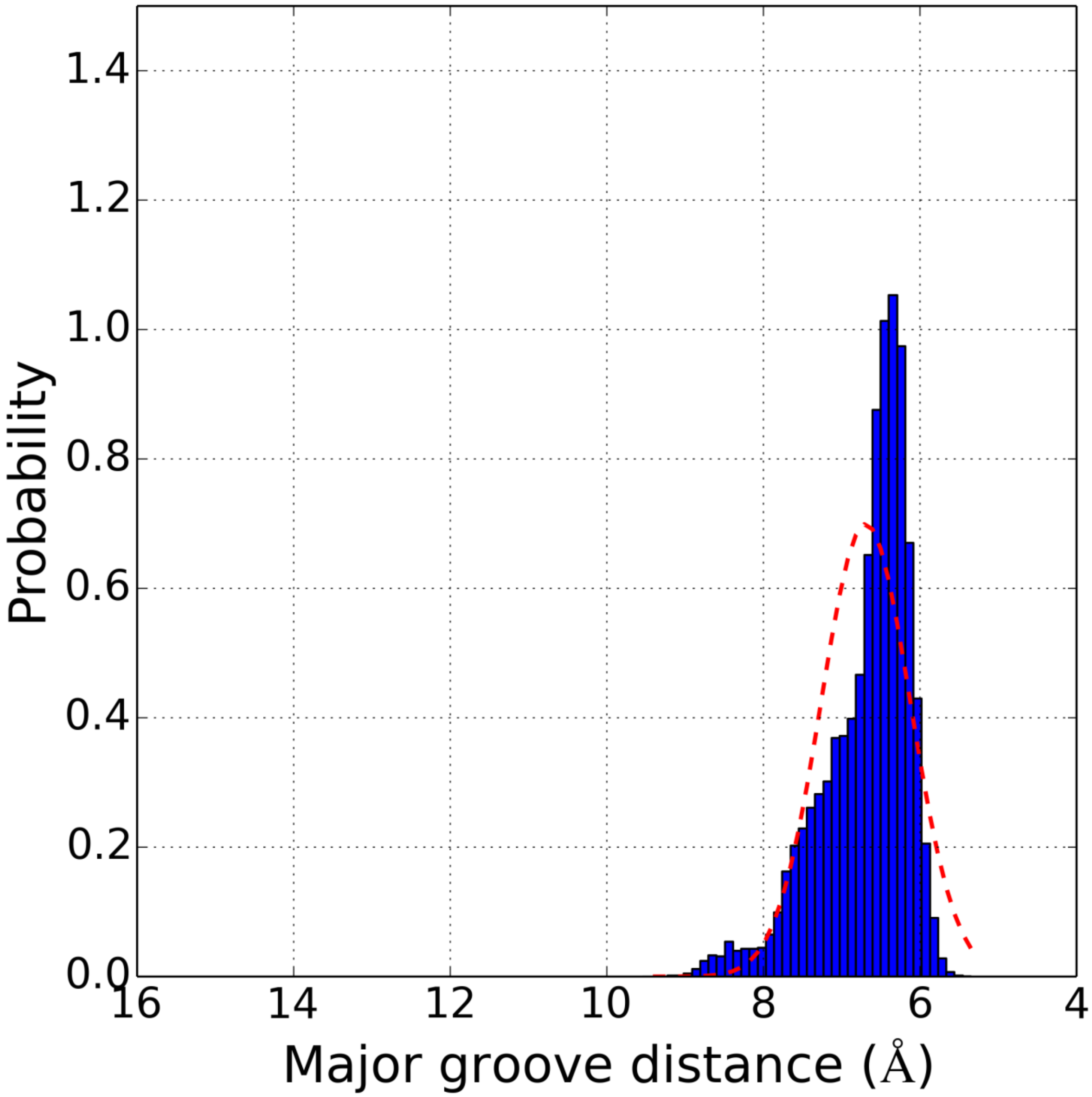

Figure S5. The expected values of our new major groove-binding distance was established by analysing the probability density of the average major groove distance between each superhelical residue and its nearest major groove site for control simulations of the specific MTERF1-DNA complex. 40 bins were used (the integral of the bins is one).

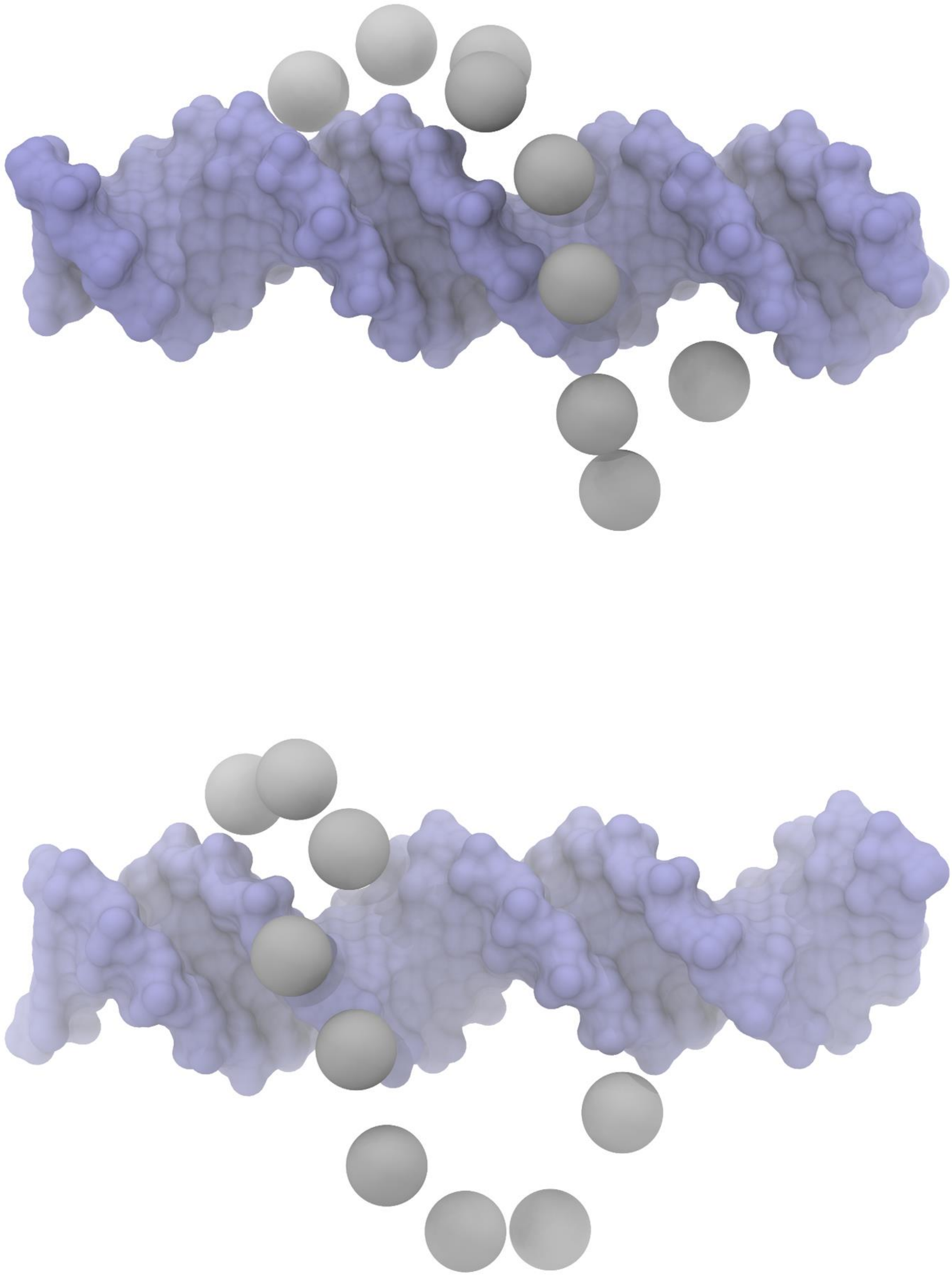

Figure S6. Quantifying the position of MTERF1 in the major groove of B-DNA. The upper pose has a groove tracking distance of 9.55 Å and the lower pose is 14.03 Å. In the upper pose, the first superhelical residue is paired to the tenth major groove site, the second superhelical residue the ninth site and so on, the individual

distances of which are 13.5, 11.1, 6.0, 8.3, 5.4, 9.2, 12.1, 14.5, and 5.9 Å. For the lower pose, the first superhelical residue is paired with the fourteenth major groove site, the second superhelical residue with the thirteenth major groove site and so on, the individual distances of which are 11.2, 11.6, 10.6, 13.5, 13.6, 17.0, 16.8, 15.9, and 16.3 Å.

SECTION 7. EQUILIBRATION DETAILS

Table S2. Equilibration procedure for explicit solvent MD simulations.

Stage	Ref	EOM	Steps (x 10^3)	Temp (K)	Ensemble	Group	Force constant (kcal/molA^2)
1	xtal	min	10	-	-	A	100
2	1	MD	100	100/300	NVT	A	100
3	2	MD	100	300	NPT	A	100
4	3	MD	250	300	NPT	A	10
5	4	min	10	-	-	B	10
6	5	MD	100	300	NPT	B	10
7	6	MD	100	300	NPT	B	1
8	7	MD	100	300	NPT	B	0.1
9	-	MD	2500	300	NPT	-	0

Ref, reference coordinates. **EOM**, equation of motion: min, minimization; MD, molecular dynamics. **Ensemble**: NPT and NVT used a weak temperature coupling thermostat with isotropic position scaling. **Group**, atoms restrained to reference structure (**Ref**): **A** for apo MTERF1, all protein heavy atoms except the sidechains of residues 1 and 2, **B** for apo MTERF1, all protein backbone atoms – Cα, N, and C; **A** for holo MTERF1, all protein and DNA heavy atoms except, as in apo MTERF1, the side chains of residues 1 and 2, **B** for holo MTERF1, all protein and DNA backbone atoms – C1’, C2’, C3’, C4’, O4’, C5’, O3’,O5’,OP1, OP2, P; **A** for search mode MTERF1, superhelical Cα atoms and all DNA heavy atoms, **B** for search mode MTERF1, only DNA backbone atoms (MTERF1 fully unrestrained). **Force constant**, harmonic force constant for Cartesian restraints. For search mode, force constants were 1/10th of those illustrated above, except for stage 8, which had no force applied. The Berendsen thermostat (19) was used for all stages of MD including production. Stage 2 and 3 used bath coupling constants of 0.1 ps; all subsequent stages of MD including production used 0.5 ps coupling constants.

SECTION 8. DOCKING B-DNA TO MTERF1 FROM THE RECOGNITION STRUCTURE

To determine whether the conformation of MTERF1 in recognition mode (the crystal structure) could bind B-DNA, a docking procedure followed by MD was used to evaluate the energetics of the complex. All poses in which B-DNA was docked into the binding cleft of MTERF1 were tested for stability using our fully atomistic MD procedure (see **Section 7**) and found to be energetically unstable (>10^8 kcal/mol). Some poses appear reasonable (**Figure S7A** and **B**) and indeed MD energies were stable, though not structurally stable (RMSD > 7 Å after only 50 ns). Closer inspection of these poses reveals that the C-site of the DNA would clash if extended. To show these poses were not simply artefacts of using short DNA, the DNA in all the poses were lengthened and the systems

subjected to the same equilibration procedure. As expected, every single docked poses was not energetically viable (>10^8 kcal/mol) due to van der Waals clashes.

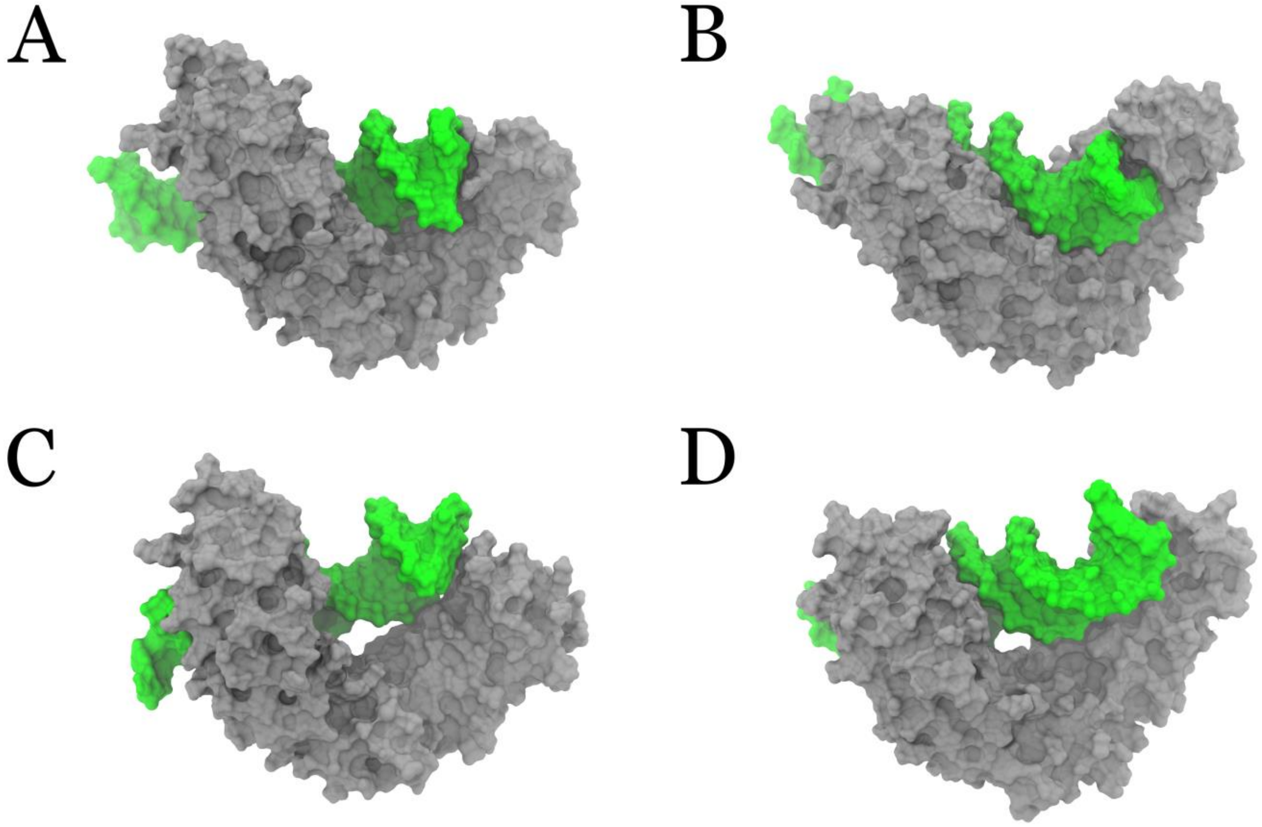

Figure S7. Docking B-DNA to MTERF1 from the recognition mode structure fails to produce poses in which the protein tracks the major groove. (**A**) One of two DOT2.0 docked poses in which MD equilibration energy did not result in high energy (>10^9 kcal/mol) using the exact procedure used to dock low-pitch apo MTERF1 to B-DNA and to dock MTERF1 from recognition mode to the corresponding unwound DNA from the crystal structure. (**B**) The second pose. (**C**) After 75 ns of unrestrained MD of the pose from (**A**), MTERF1 dissociates from the DNA (all atom RMSD > 7 Å) (**D**) After 50 ns of unrestrained MD of the pose from (**B**), MTERF1 dissociates from the DNA (all atom RMSD > 8 Å).

SECTION 9. RMSD ANALYSIS OF CONTROL AND APOMTERF1 SIMULATIONS

To establish a baseline for the apo MTERF1 conformational change, 4 independent 1.5 µs control simulations of the MTERF1-DNA specific complex were performed. Small structural fluctuations were expected and the conformation of MTERF1 would on average be similar to the crystal structure. To quantify how similar our MD structures were to the reference crystal structure, the root mean square positional deviations (RMSD) between our MD snapshots and the equilibrated crystal structure were measured using cpptraj (20). The terminal nucleotides (three per strand, for both 5’ and 3’ ends) were excluded to avoid overweighting deviations arising

from end-fraying (21). The evolution of RMSD in the control simulations displayed in **Figure S8A** shows that the conformation of MTERF1 throughout the simulations remains similar to that of the reference. In recognition mode, MTERF1 and the DNA remain tightly bound with relatively little conformational fluctuation compared to apo MTERF1.

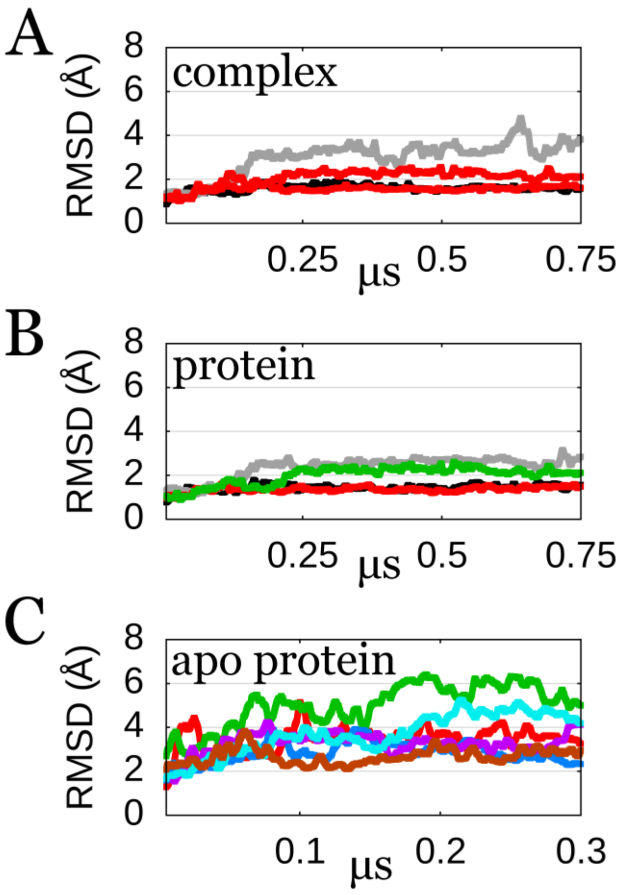

Figure S8. RMSD analysis of apo MTERF1 and holo MTERF1 (specific complex) unrestrained MD simulations. (**A**) RMSD of the specific MTERF1-DNA complex (crystal structure) protein backbone, excluding the first *mterf* motif and the C-tail, and the DNA C1' and P atoms, excluding the 3 terminal base pairs at each end. (**B**) RMSD of only the protein in the specific complex using the same atoms as in (**A**). (**C**) RMSD of MTERF1 in the unbound protein simulations using the same atoms as in (**A**) and (**B**).

The RMSD was also used as a more routine metric of conformational heterogeneity to help gauge the degree to which our helical analyses resolve the hypothesized apo MTERF1 conformational change. Thus the time-resolved RMSD of apo MTERF1 is reported in **Figure S8C**. The analysis highlights the potential problems of a crude metric of conformational such as RMSD. Simulations in which the RMSD was lower than other simulations never sampled structures with superhelical pitch < 42 Å while simulations with high RMSD never sampled low superhelical pitch.

SECTION 10. THE SIMILARITY OF ANM AND MD LOWEST FREQUENCY MOTIONS

Table S3. ANM and MD eigenvector RMSIP similarity analysis

Root mean square inner product (RMSIP)(22) of all top ten eigenvectors (1 to 10), the top four eigenvectors (1 to 4), the top three (1 to 3), and the top two (1 to 2). RMSIP provides a global similarity comparison of eigenvector overlaps, accounting for the possibility that corresponding MD and ANM eigenvectors are not in the same order. The eigenvectors defining each of the ANM modes and MD PCs would be parallel if they were identical and orthogonal if completely unrelated; the dot product of parallel vectors is zero if they are orthogonal and one if they are parallel.

		MD (PC)			
		**1 to 2**	**1 to 3**	**1 to 4**	**1 to 10**
ANM (mode)	**1 to 2**	0.776			
	**1 to 3**		0.879		
	**1 to 4**			0.856	
	**1 to 10**				0.769

SECTION 11. HISTOGRAMS OF HOLO MTERF1 AND APO MTERF1 HELICAL PARAMETERS

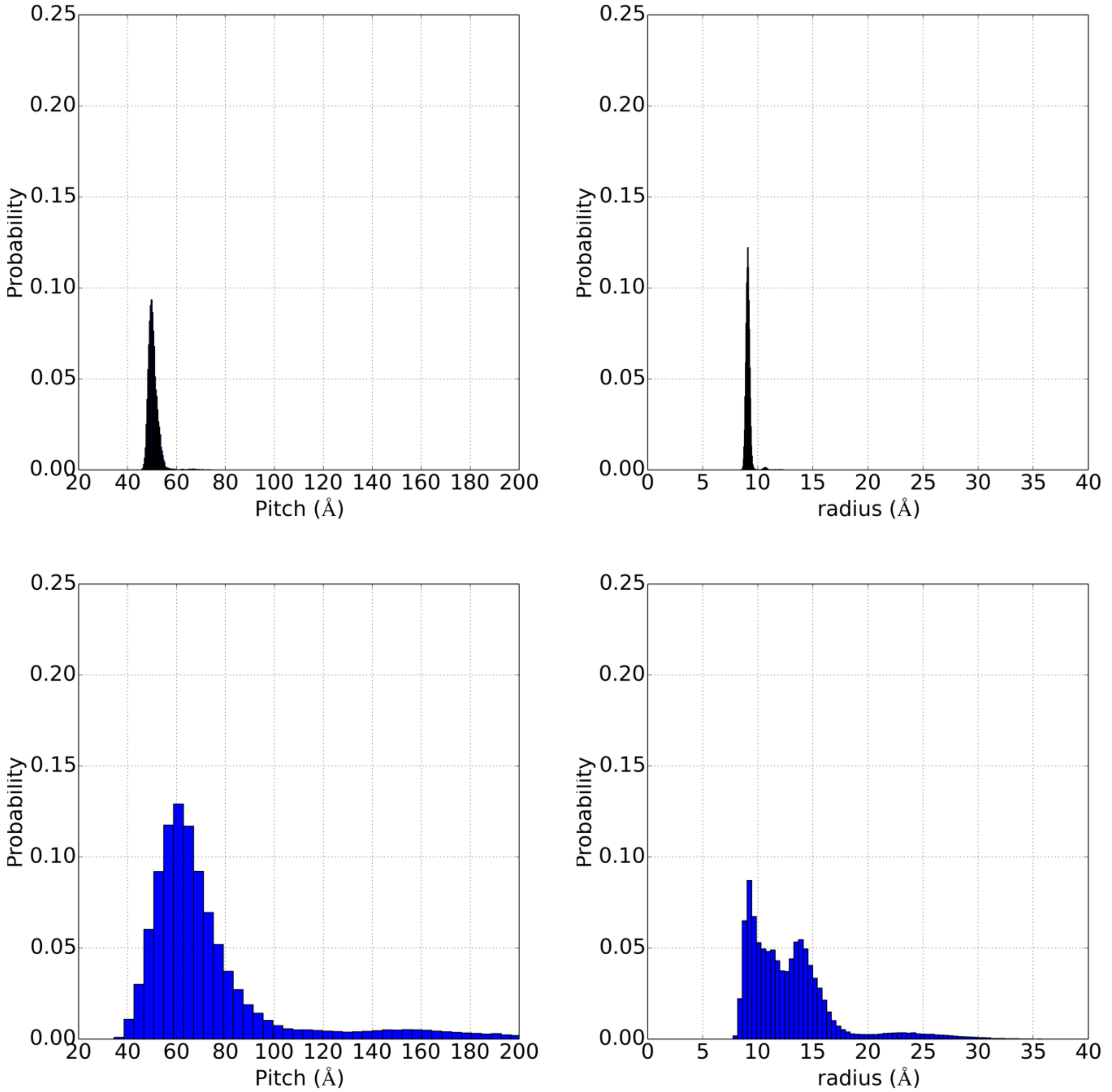

Figure S9. Histograms of helical parameters of holo and apo MTERF1 MD ensembles. Probabilities of holo MTERF1 pitch (top left), holo MTERF1 radius (top right), apo MTERF1 pitch (bottom left), and apo MTERF1 radius (bottom right).

SECTION 12. PRODUCTIVE, ENERGETICALLY STABLE NONSPECIFIC DOCKED COMPLEXES

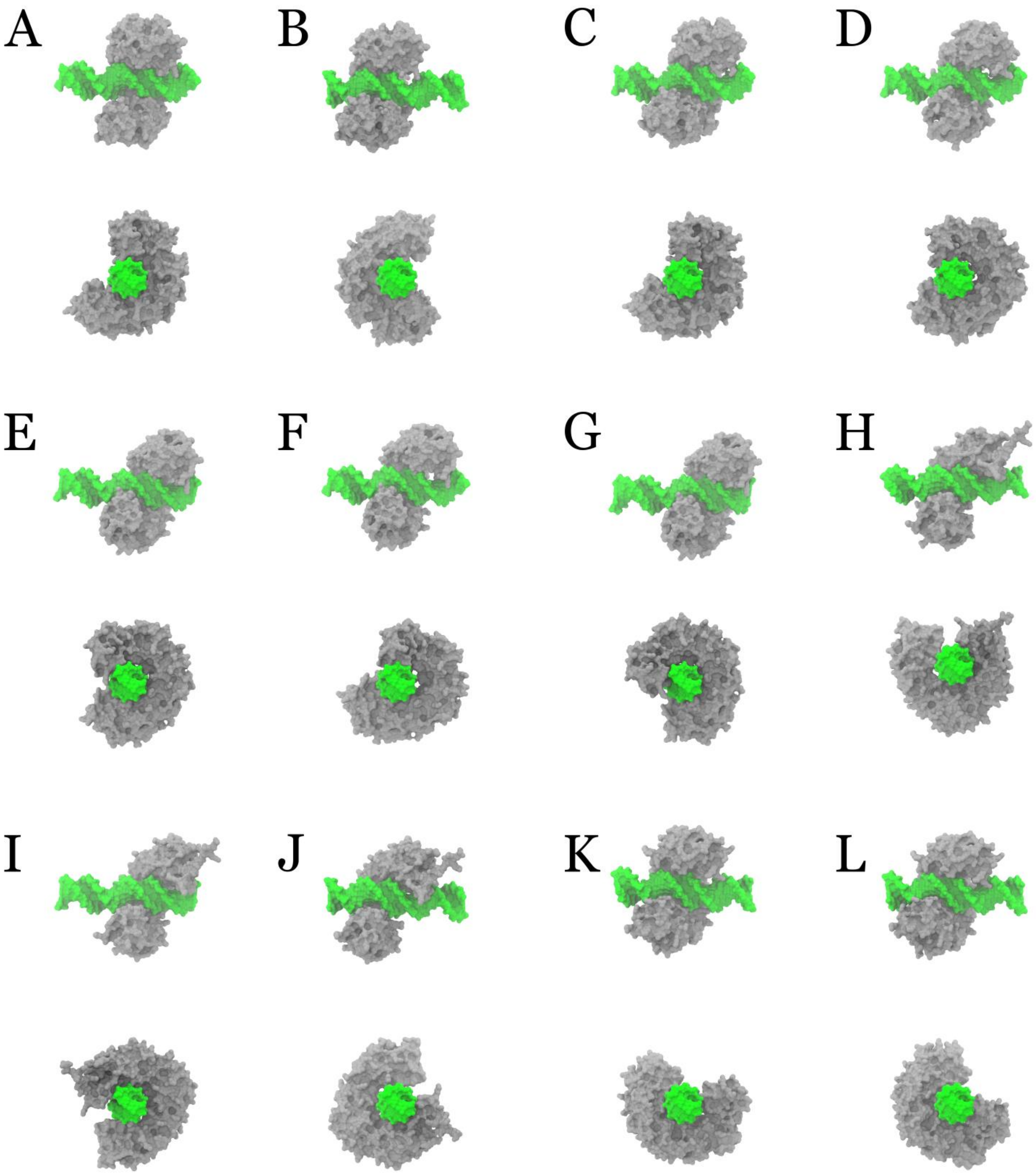

Figure S10. The 12 models of the nonspecific complex that best track the major groove obtained by docking low-pitch apo MTERF1 structures to B-DNA. The protein conformations that were used to generate the pose has the following helical parameters (pitch, radius, sweep): for (**A**) and (**B**): 41.1 Å, 9.8 Å, 372°; for (**C**): 41.6 Å, 10.1 Å,

368°; for (**D**), (**E**), (**F**) and (**G**): 40.3 Å, 11.4 Å, 340°; for (**H**) and (**I**): 39.8 Å, 16.2 Å, 276°; for (**J**): 37.8 Å, 16.0 Å, 287°; for (**K**) and (**L**): 34.5 Å, 9.8 Å, 389°.

SECTION 13. QUANTIFYING HOW TIGHTLY MTERF1 BINDS DNA

To complement the analysis in **Section 12**, the area shared by MTERF1 and DNA was evaluated using the Linear Combination of Pair Overlaps (LCPO) method (23). Since we were not calculating forces, the non-recursive LCPO algorithm (less accurate than the recursive algorithm used in GB simulations) was sufficient. The cpptraj module in AmberTools (20) was used to perform the calculation and a bash script to post-process the three sets of surface areas – one half of the protein surface area plus one half of the DNA surface area minus one half of the complex surface area.

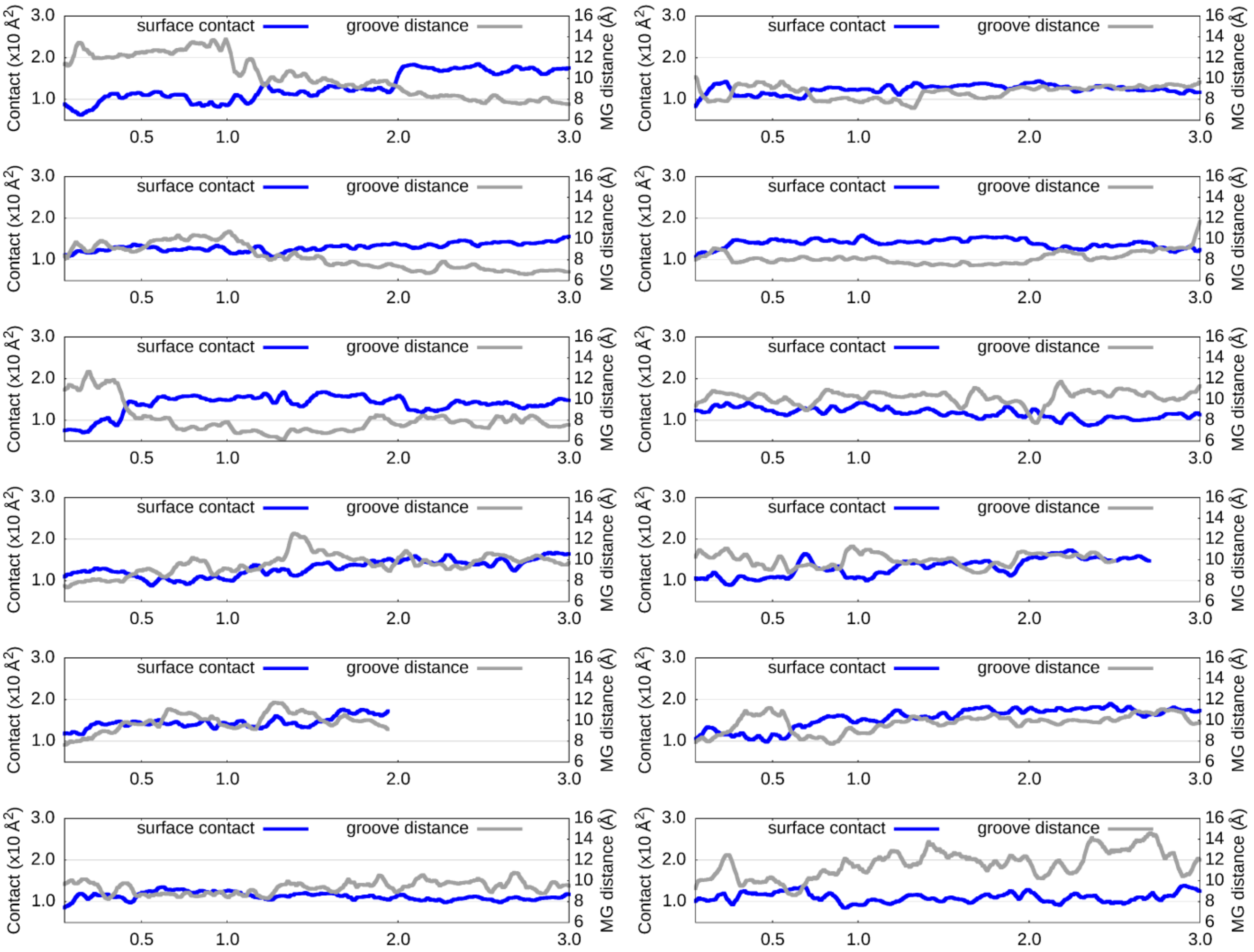

Figure S11. The shared surface area of MTERF1 and DNA in the 12 distinct search mode complexes. The abscissa is time, in units of μs.

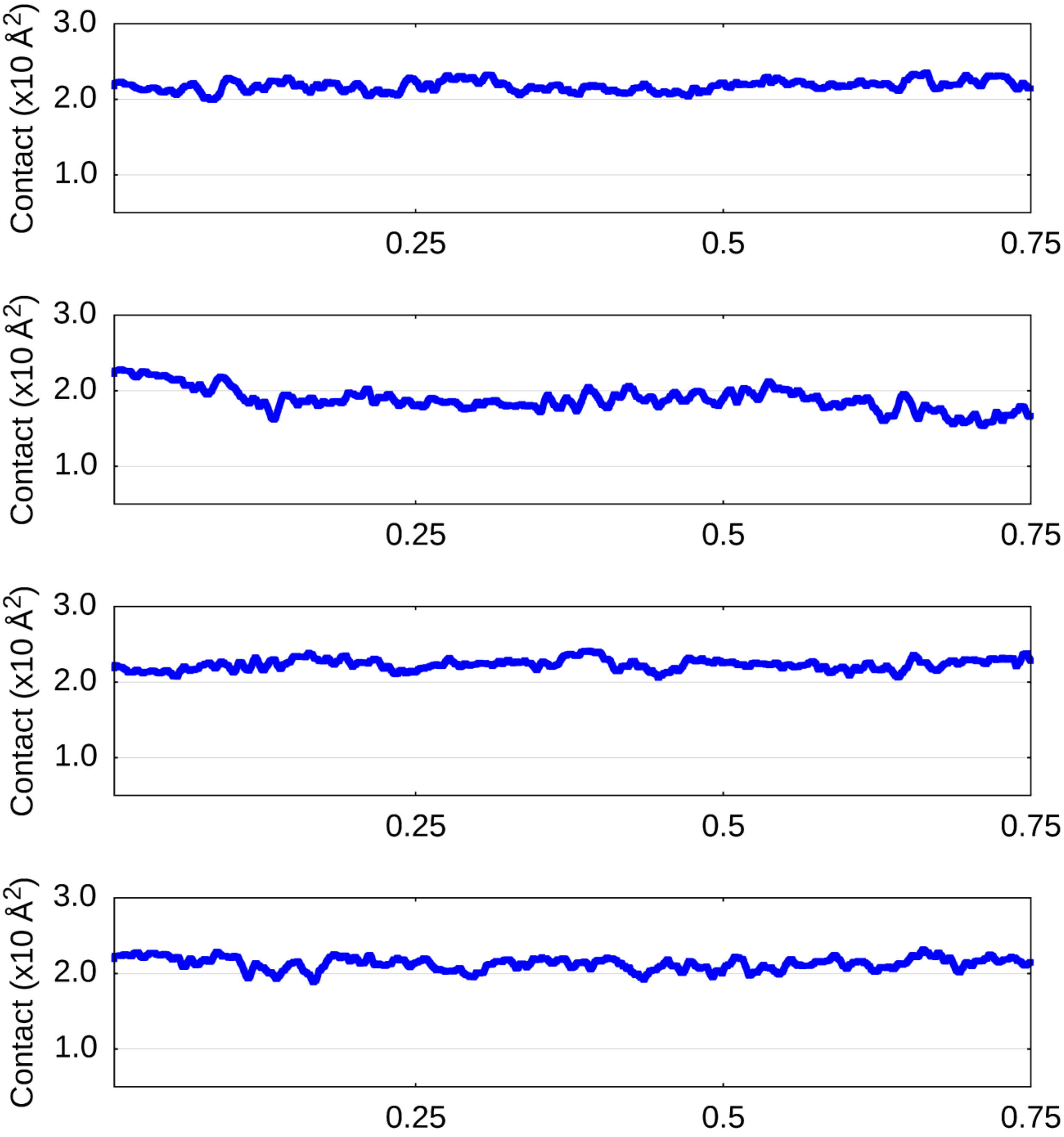

Figure S12. The shared surface area of MTERF1 and DNA in the recognition complex for each of the four independent simulations. The abscissa is time, in units of μs.

SECTION 14. MEASURING MTERF1 TRANSLOCATION ALONG DNA

To quantify MTERF1 sliding a similar approach to that used by the Levy lab (24) was used. The metric was used to distinguish geometries that were sliding, hopping, or freely diffusing. Here, the measurement has a specific meaning here for two reasons. First, MTERF1 wraps around the DNA, so the protein centre-of-mass (COM) falls near the DNA axis. Second, we use the sliding distance in combination with our major groove-tracking distance to ensure the sliding distance is reporting on translocation and not unbinding.

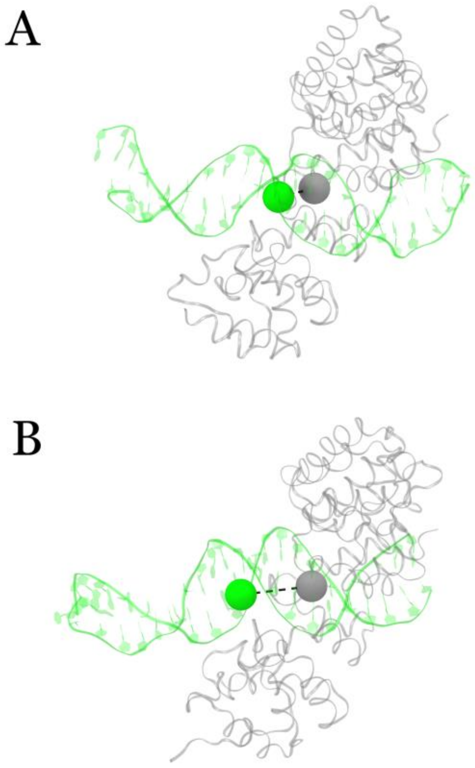

Figure S13. Visualization of our sliding distance metric. The protein (grey) and DNA (green) are shown as translucent cartoons to highlight the centres-of-mass. The centre-of-mass (COM) of the superhelical residues is shown as a grey sphere. The COM of the C1' atoms in the DNA, excluding 4 bases at each end of the duplex to prevent end-fraying artefacts, is shown as a green sphere. (**A**) The equilibrated snapshot (0.5 µs) of the search mode. (**B**) A snapshot of the search mode (1.4 µs) that shows increased distance between the COM of the protein and the DNA.

REFERENCES

1. Yakubovskaya, E., Mejia, E., Byrnes, J., Hambardjieva, E. and Garcia-Diaz, M. (2010) Helix Unwinding and Base Flipping Enable Human MTERF1 to Terminate Mitochondrial Transcription. *Cell*, **141**, 982-993.
2. Lavery, R., Moakher, M., Maddocks, J.H., Petkeviciute, D. and Zakrzewska, K. (2009) Conformational analysis of nucleic acids revisited: Curves. *Nucleic Acids Res*, **37**, 5917-5929.
3. Whitworth, W.A. (1875) The regular polygon in space. *Messenger of Mathematics*, **4**, 88-89.

4. Cochran, W., Crick, F. and Vand, V. (1952) The structure of synthetic polypeptides. I. The transform of atoms on a helix. *Acta Crystallographica*, **5**, 581-586.
5. Christopher, J.A., Swanson, R. and Baldwin, T.O. (1996) Algorithms for finding the axis of a helix: fast rotational and parametric least-squares methods. *Computers & chemistry*, **20**, 339-345.
6. Nievergelt, Y. (1997) Fitting helices to data by total least squares. *Computer aided geometric design*, **14**, 707-718.
7. Krivoshapko, S.N. and Ivanov, V.N. *Encyclopedia of analytical surfaces*.
8. Eyal, E., Yang, L.W. and Bahar, I. (2006) Anisotropic network model: systematic evaluation and a new web interface. *Bioinformatics*, **22**, 2619-2627.
9. Macke, T.J. and Case, D.A. (1998) Modeling unusual nucleic acid structures. *Molecular Modeling of Nucleic Acids*, **682**, 379-393.
10. Pasi, M., Maddocks, J.H., Beveridge, D., Bishop, T.C., Case, D.A., Cheatham, T., Dans, P.D., Jayaram, B., Lankas, F., Laughton, C., Mitchell, J., Osman, R., Orozco, M., Pérez, A., Petkevičiūtė, D., Spackova, N., Sponer, J., Zakrzewska, K. and Lavery, R. (2014) μABC: a systematic microsecond molecular dynamics study of tetranucleotide sequence effects in B-DNA. *Nucleic Acids Research*.
11. Pedone, F. and Santoni, D. (2009) Sequence-dependent DNA helical rise and nucleosome stability. *BMC Mol Biol*, **10**, 105.
12. Pasi, M., Maddocks, J.H., Beveridge, D., Bishop, T.C., Case, D.A., Cheatham, T., 3rd, Dans, P.D., Jayaram, B., Lankas, F., Laughton, C., Mitchell, J., Osman, R., Orozco, M., Perez, A., Petkeviciute, D., Spackova, N., Sponer, J., Zakrzewska, K. and Lavery, R. (2014) muABC: a systematic microsecond molecular dynamics study of tetranucleotide sequence effects in B-DNA. *Nucleic Acids Res.*, **42**, 12272-12283.
13. Olson, W.K., Gorin, A.A., Lu, X.J., Hock, L.M. and Zhurkin, V.B. (1998) DNA sequence-dependent deformability deduced from protein-DNA crystal complexes. *Proc Natl Acad Sci U S A*, **95**, 11163-11168.
14. Roberts, V.A., Pique, M.E., Ten Eyck, L.F. and Li, S. (2013) Predicting protein-DNA interactions by full search computational docking. *Proteins-Structure Function and Bioinformatics*, **81**, 2106-2118.
15. Roberts, V.A., Case, D.A. and Tsui, V. (2004) Predicting interactions of winged-helix transcription factors with DNA. *Proteins-Structure Function and Bioinformatics*, **57**, 172-187.
16. Word, J.M., Lovell, S.C., LaBean, T.H., Taylor, H.C., Zalis, M.E., Presley, B.K., Richardson, J.S. and Richardson, D.C. (1999) Visualizing and quantifying molecular goodness-of-fit: small-probe contact dots with explicit hydrogen atoms. *J Mol Biol*, **285**, 1711-1733.
17. Baker, N.A., Sept, D., Joseph, S., Holst, M.J. and McCammon, J.A. (2001) Electrostatics of nanosystems: application to microtubules and the ribosome. *Proc Natl Acad Sci U S A*, **98**, 10037-10041.
18. Sanner, M.F., Olson, A.J. and Spehner, J.C. (1996) Reduced surface: an efficient way to compute molecular surfaces. *Biopolymers*, **38**, 305-320.
19. Berendsen, H.J.C., Postma, J.P.M., van Gunsteren, W.F., DiNola, A. and Haak, J.R. (1984) Molecular dynamics with coupling to an external bath. *The Journal of chemical physics*, **81**, 3684-3690 %@ 0021-9606.
20. Roe, D.R. and Cheatham, T.E. (2013) PTRAJ and CPPTRAJ: Software for Processing and Analysis of Molecular Dynamics Trajectory Data. *J Chem Theory Comput*, **9**, 3084-3095.
21. Galindo-Murillo, R., Roe, D.R. and Cheatham, T.E., 3rd. (2014) On the absence of intrahelical DNA dynamics on the mus to ms timescale. *Nat Commun*, **5**, 5152.
22. Skjaerven, L., Martinez, A. and Reuter, N. (2011) Principal component and normal mode analysis of proteins; a quantitative comparison using the GroEL subunit. *Proteins*, **79**, 232-243.
23. Weiser, J., Shenkin, P.S. and Still, W.C. (1999) Approximate atomic surfaces from linear combinations of pairwise overlaps (LCPO). *Journal of Computational Chemistry*, **20**, 217-230.
24. Bhattacherjee, A. and Levy, Y. (2014) Search by proteins for their DNA target site: 2. The effect of DNA conformation on the dynamics of multidomain proteins. *Nucleic Acids Research*, **42**, 12415-12424.
